# Supplementary material for: Covalent Adducts Formed by the Androgen Receptor Transactivation Domain and Small Molecule Drugs Remain Disordered
Source: J Chem Inf Model. 2025 May 30;65(12):6221–37. doi: 10.1021/acs.jcim.5c00833 (PMC12199303; doi:10.1021/acs.jcim.5c00833)
Supplement: Supplementary file 3 [file ci5c00833_si_004.pdf]

Covalent adducts formed by the androgen receptor transactivation domain and small molecule drugs remain disordered

Jiaqi Zhu and Paul J. Robustelli*

Department of Chemistry, Dartmouth College, Hanover, NH, 03755, USA

E-mail: paul.j.robustelli@dartmouth.edu

Supporting Information

MD Simulation Convergence Analyses

The convergence of each covalent adduct simulation was assessed by comparing the and secondary structure propensities and the populations of intramolecular protein-ligand contacts for each temperature rung in the REST2 simulation in SI Figure S1, SI Figure S3, SI Figure S5 and SI Figure S7. The relative smooth temperature dependence of these properties suggest that simulations are reasonably well converged. The same analyses were performed on the demultiplexed replicas, which follow each independent replica through temperature space, to determine if any individual replicas became stuck in local minima as they diffuse through the temperature ladder in SI Figure S2, SI Figure S4, SI Figure S6, and SI Figure S8. We found the statistical fluctuations to be reasonably well converged among demultiplexed replicas.

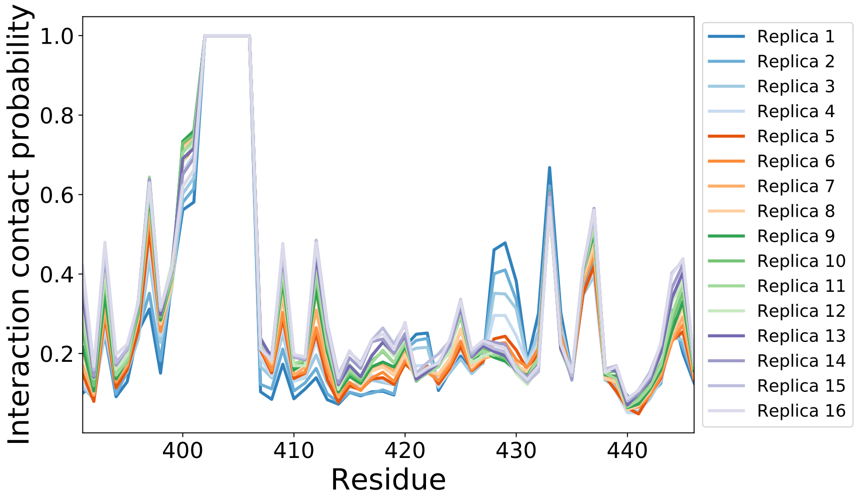

Figure S1: Intramolecular protein-ligand contacts observed in a REST2 MD simulation of Tau-5$_{\text{R2_R3}}$-CYS404:EPI-002. Intramolecular contact probabilities between the covalently modified CYS404:EPI-002 residue and Tau-5$_{\text{R2_R3}}$ residues observed in the 16 solute temperature rungs spanning 300K-500K. Contacts between CYS404:EPI-022 and Tau-5$_{\text{R2_R3}}$ residues are defined using a cutoff of 6Å between heavy atoms.

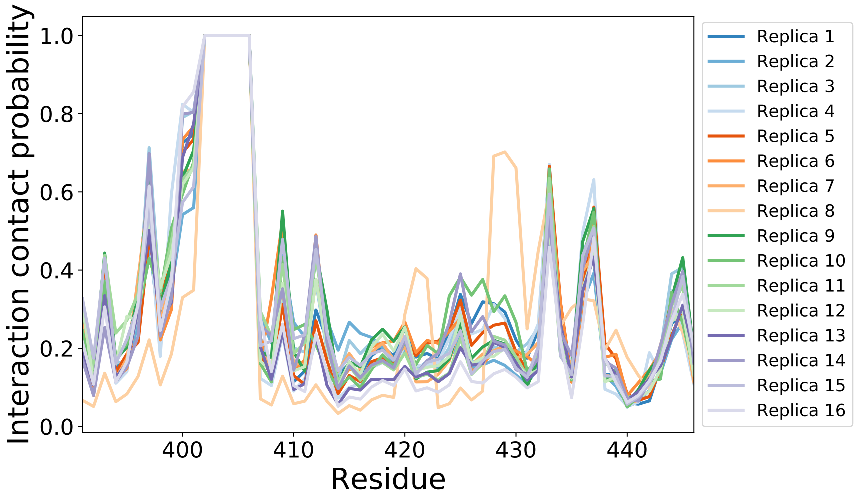

Figure S2: Intramolecular protein-ligand contacts observed in the demultiplexed replicas of a REST2 MD simulation of Tau-5$_{\text{R2_R3}}$-CYS404:EPI-002. Intramolecular contact probabilities between the covalently modified CYS404:EPI-002 residue and Tau-5$_{\text{R2_R3}}$ residues observed in the 16 demultiplexed replicas. Contacts between CYS404:EPI-002 and Tau-5$_{\text{R2_R3}}$ residues are defined using a cutoff of 6Å between heavy atoms.

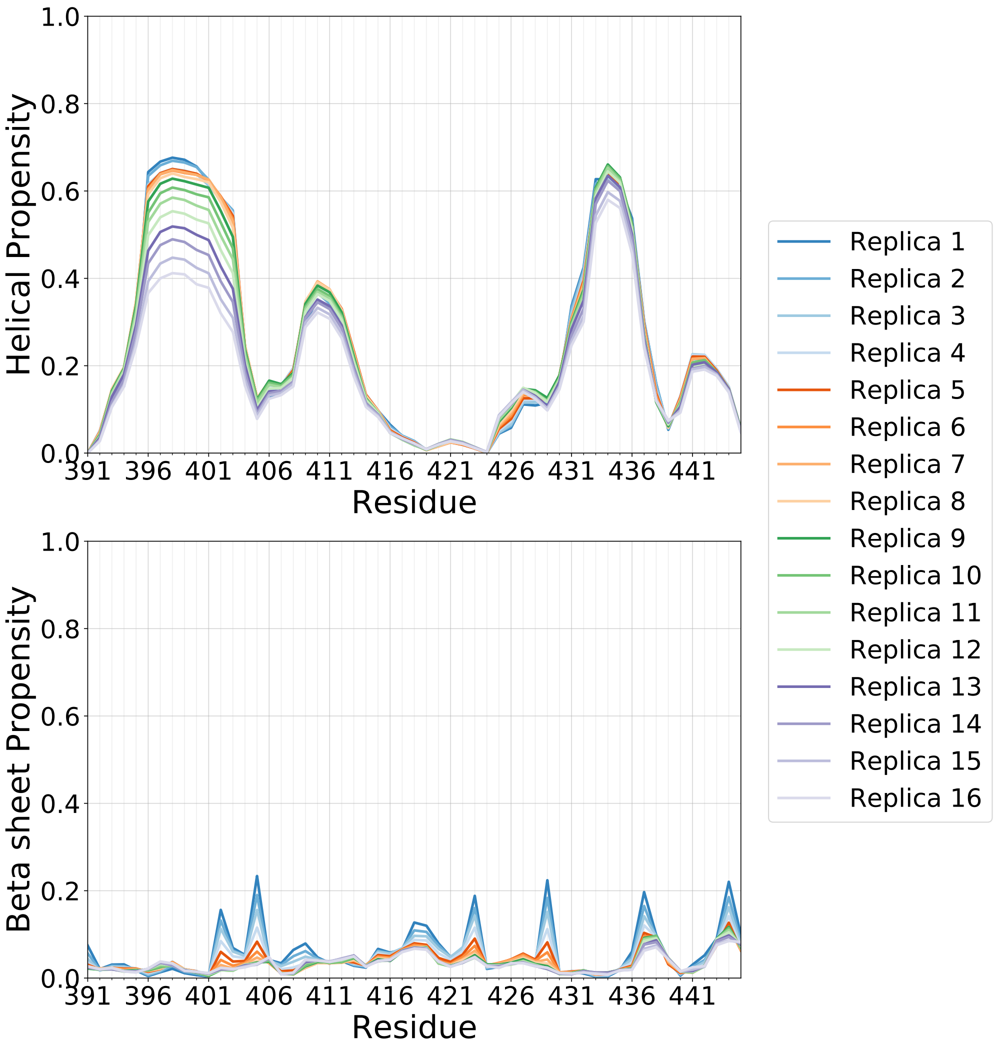

Figure S3: Secondary structure propensities observed in a REST2 MD simulation of Tau-5$_{\mathbf{R2_R3}}$-CYS404:EPI-002. Comparison of α-helical and β-sheet propensities of Tau-5$_{\mathrm{R2_R3}}$ observed in the 16 solute temperature rungs spanning 300K-500K. Secondary structure content is calculated by the DSSP algorithm.

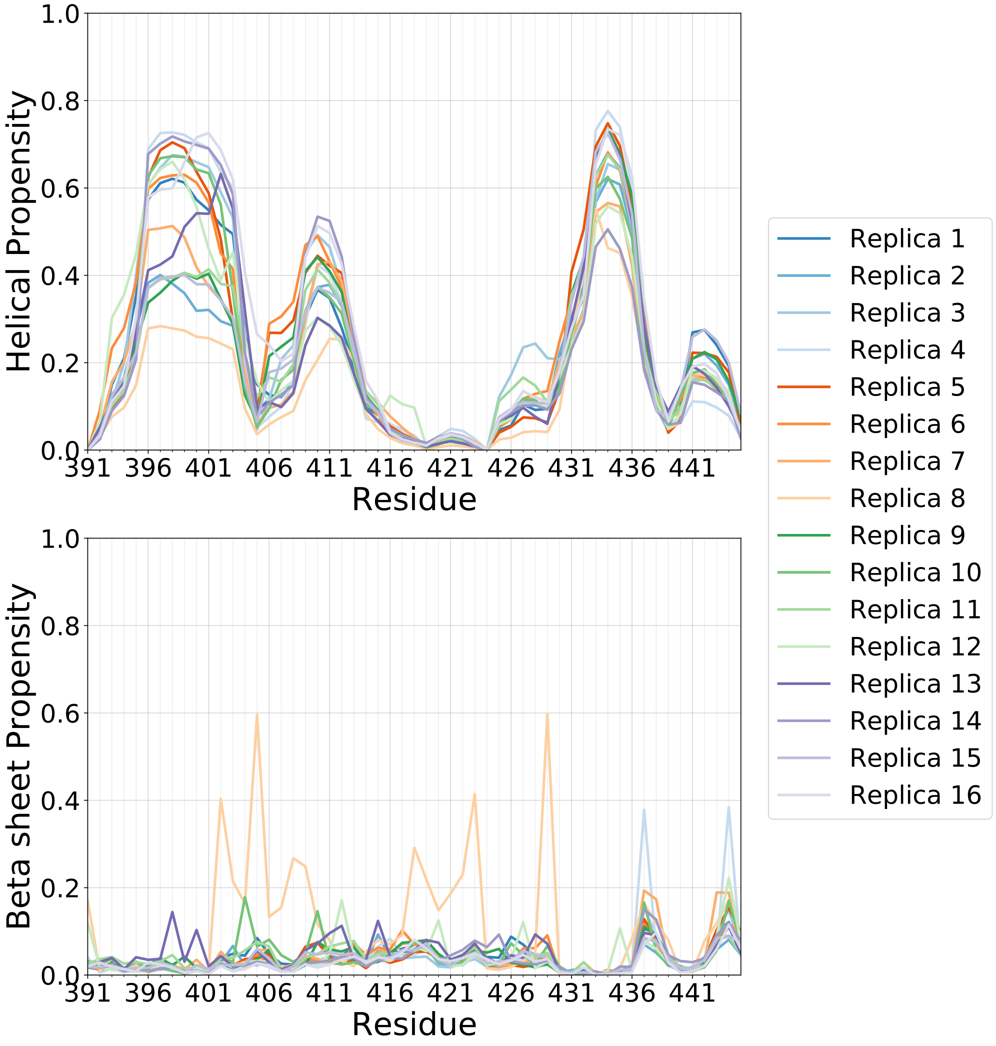

Figure S4: Secondary structure propensities observed in the demultiplexed replicas of a REST2 MD simulation of Tau-5$_{\mathbf{R2_R3}}$-CYS404:EPI-002. Comparison of α-helical and β-sheet propensities of Tau-5$_{\mathrm{R2_R3}}$ observed in the 16 demultiplexed replicas of a REST2 MD simulation of Tau-5$_{\mathrm{R2_R3}}$-CYS404:EPI-002. Secondary structure content is calculated by the DSSP algorithm. Replica 8 has the largest deviations from the average secondary structure propensities of the demultiplexed replicas.

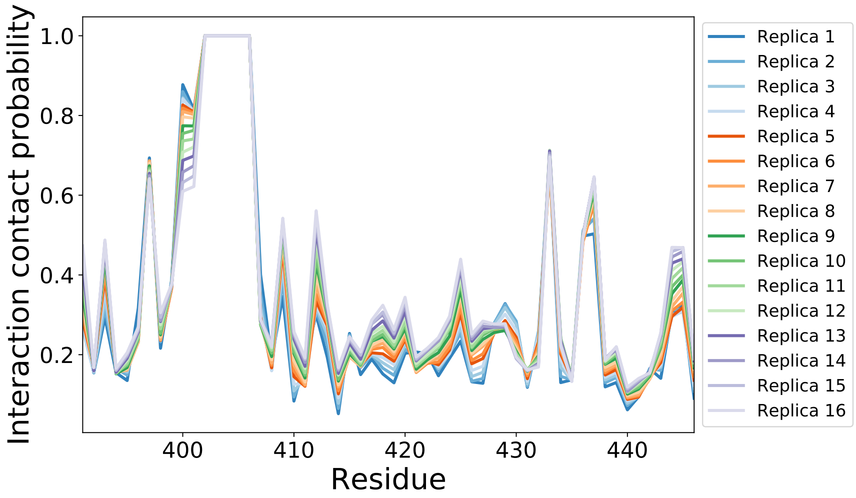

Figure S5: Intramolecular protein-ligand contacts observed in a REST2 MD simulation of Tau-5$_{\mathbf{R2_R3}}$-CYS404:EPI-7170. Intramolecular contact probabilities between the covalently modified CYS404:EPI-7170 residue and Tau-5$_{R2_R3}$ residues observed in the 16 solute temperature rungs spanning 300K-500K. Contacts between CYS404:EPI-7170 and Tau-5$_{R2_R3}$ residues are defined using a cutoff of 6Å between heavy atoms.

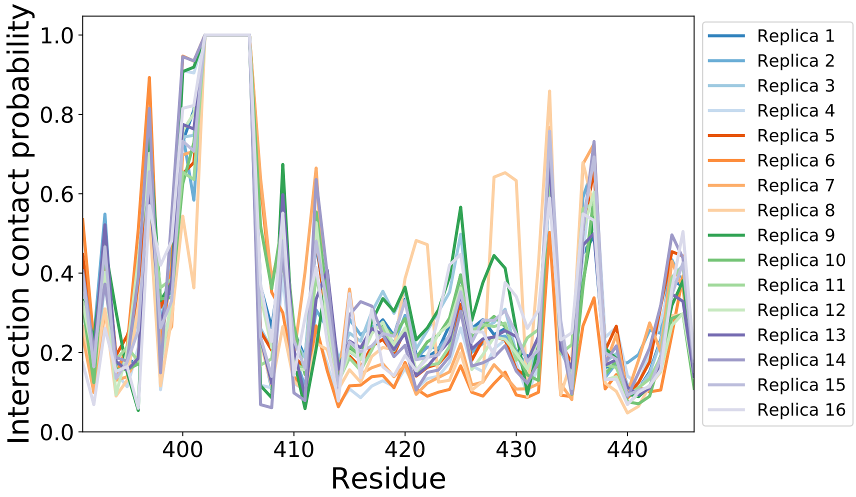

Figure S6: Intramolecular protein-ligand contacts observed in the demultiplexed replicas of a REST2 MD simulation of Tau-5$_{\mathbf{R2_R3}}$-CYS404:EPI-7170. Intramolecular contact probabilities between the covalently modified CYS404:EPI-7170 residue and Tau-5$_{R2_R3}$ residues observed in the 16 demultiplexed replicas. Contacts between CYS404:EPI-7170 and Tau-5$_{R2_R3}$ residues are defined using a cutoff of 6Å between heavy atoms.

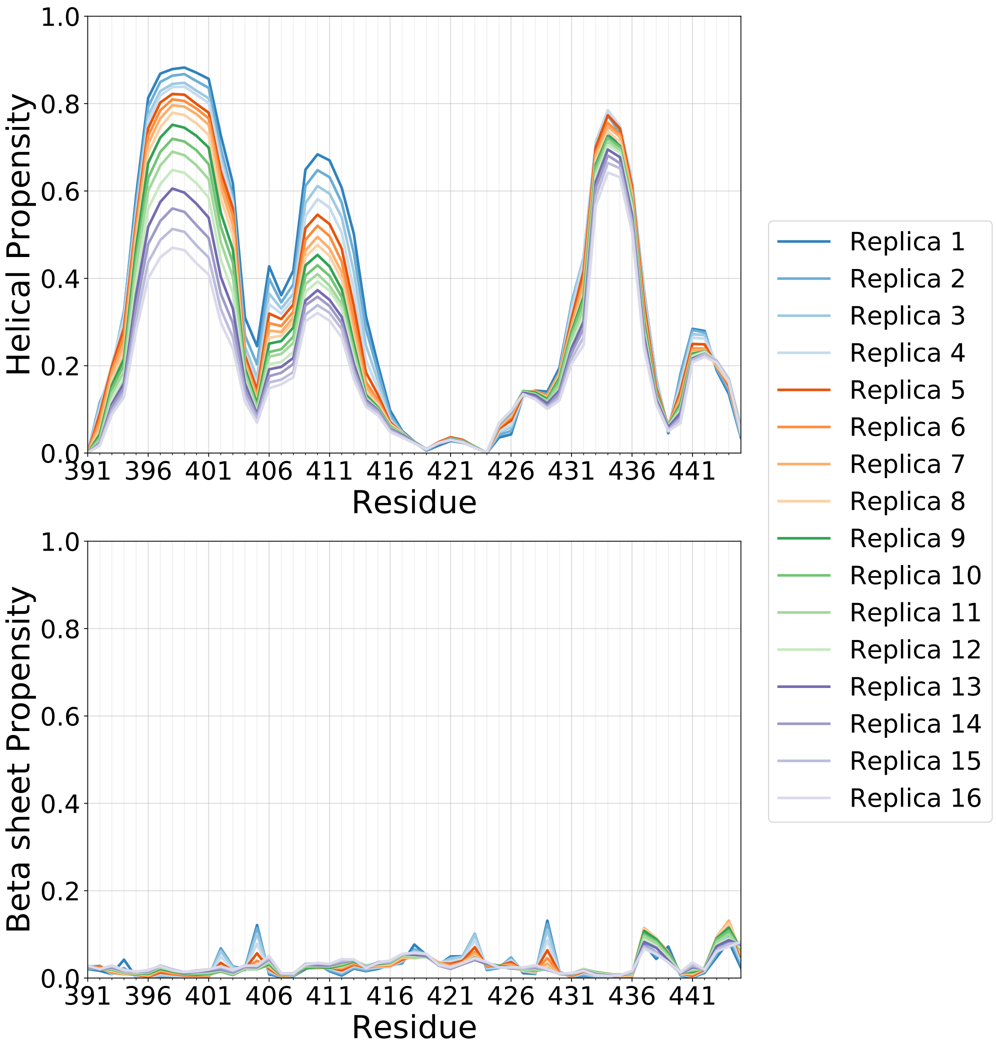

Figure S7: Secondary structure propensities observed in a REST2 MD simulation of Tau-5$_{\mathbf{R2_R3}}$-CYS404:EPI-7170. Comparison of α-helical and β-sheet propensities of Tau-5$_{\mathrm{R2_R3}}$ observed in the 16 solute temperature rungs spanning 300K-500K. Secondary structure content is calculated by the DSSP algorithm.

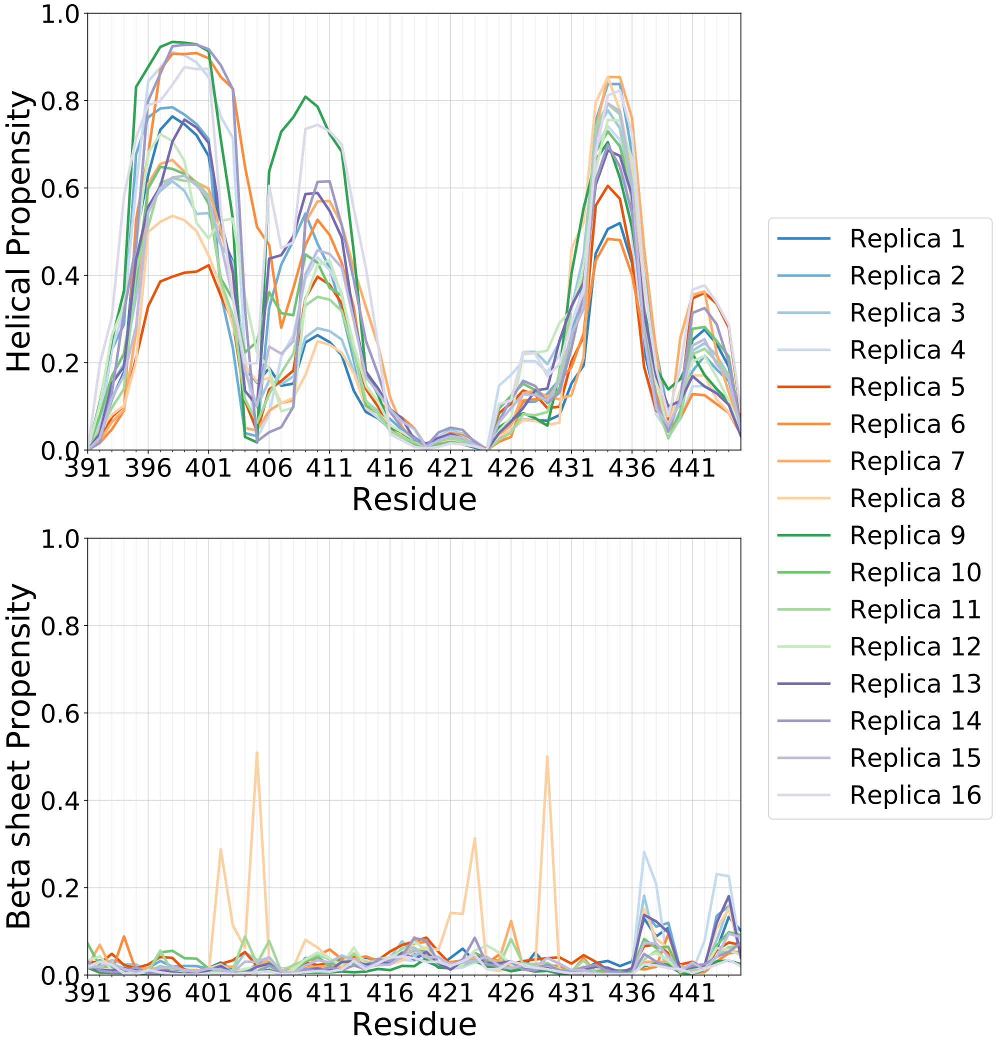

Figure S8: Secondary structure propensities observed in the demultiplexed replicas of a REST2 MD simulation of Tau-5$_{R2_R3}$-CYS404:EPI-7170. Comparison of α-helical and β-sheet propensities of Tau-5$_{R2_R3}$ observed in the 16 demultiplexed replicas of a REST2 MD simulation of Tau-5$_{R2_R3}$-CYS404:EPI-7170. Secondary structure content is calculated by the DSSP algorithm. Replica 8 has the largest deviations from the average secondary structure propensities of the demultiplexed replicas.

Table S1: Acceptance ratios for REST2 simulations of covalent adducts of Tau-5$_{\mathbf{R2_R3}}$-CYS404:EPI-002 Tau-5$_{\mathbf{R2_R3}}$-CYS404:EPI-7170 . The acceptance ratios are reported for each pair of replicas that exchange across the temperature ladder ranging from 300 K to 500 K. We observe that some temperature rungs have lower acceptance ratios in each simulation and confirm satisfactory temperature rung exploration by analyzing the temperature rung diffusion and conformational space sampled by each demultiplexed replica.

Replica	Temperature (K)	CYS404:EPI-002	CYS404:EPI-7170
1x2	300	0.03	0.17
2x3	310	0.04	0.04
3x4	321	0.33	0.25
4x5	332	0.40	0.03
5x6	344	0.41	0.17
6x7	355	0.41	0.18
7x8	368	0.34	0.41
8x9	381	0.13	0.25
9x10	394	0.42	0.25
10x11	408	0.03	0.42
11x12	422	0.35	0.43
12x13	436	0.33	0.31
13x14	451	0.36	0.07
14x15	467	0.36	0.43
15x16	483	0.34	0.03
Average		0.29	0.23

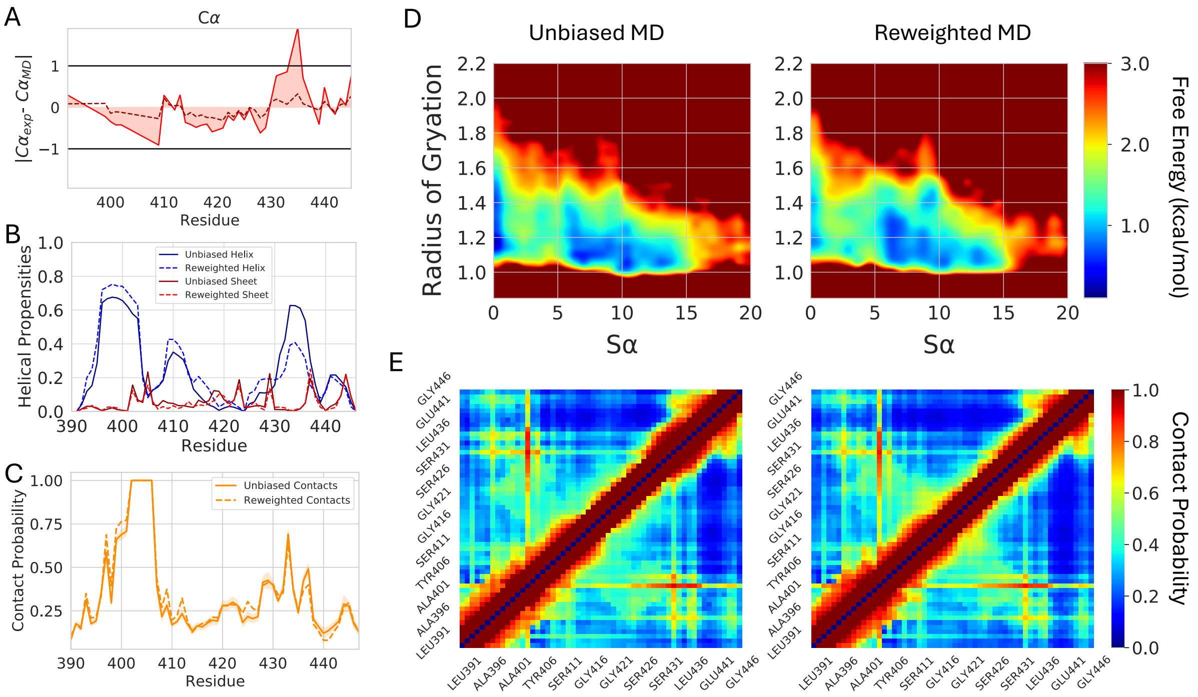

Figure S9: Comparison of experimental NMR data with molecular dynamics (MD) simulations before and after reweighting. A) Comparison of experimental NMR $C\alpha$ chemical shifts (BMRB entry 53115) with shifts calculated from MD simulations using SPARTA+ before (red) and after maximum-entropy reweighting (discontinuous line, dark red). Black lines indicate the average $C\alpha$ chemical shift prediction error of SPARTA+ on its training database of folded protein structures. **B)** Helical (blue) and Beta sheet (red) propensity of Tau-5_{R2_R3}-CYS404:EPI-002 before (solid) and after (dashed) reweighting. Secondary structure propensities were calculated using the DSSP algorithm. **C)** Intramolecular contact probabilities between covalently modified CYS404 residues and Tau-5_{R2_R3}-CYS404:EPI-002 residues. **D)** Free energy surface as a function of the radius of gyration (Rg) and $S\alpha$ before and after reweighting for Tau-5_{R2_R3}-CYS404:EPI-002. **E)** Intramolecular contact populations of covalent adduct conformational states for Tau-5_{R2_R3}-CYS404:EPI-002 before and after reweighting. Contacts between residues are defined using a distance cutoff of 12Å between closest heavy atoms.

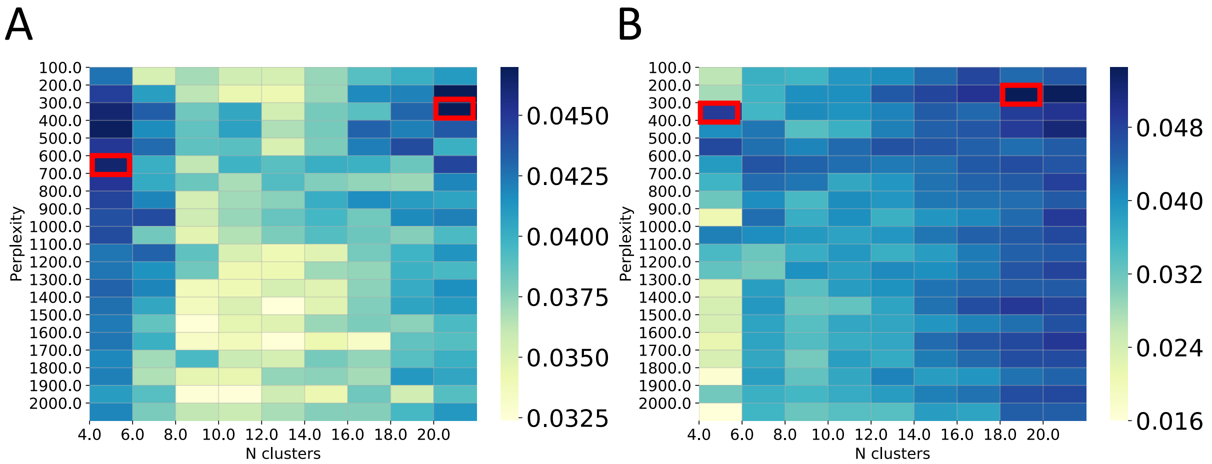

Figure S10: t-SNE clustering hyperparameter selection. Identification of optimal t-SNE clustering hyperparameters perplexity (*perp*) and number of clusters (N) for a merged ensemble containing the Tau-5$_{\mathrm{R2_R3}}$-CYS404:EPI-002 and Tau-5$_{\mathrm{R2_R3}}$-CYS404:EPI-7170 covalent adduct ensembles **(A)** and a merged ensemble containing all frames from non-covalent Tau-5$_{\mathrm{R2_R3}}$ EPI-002 and EPI-7170 ligand-binding simulations **(B)**. We perform t-SNE clustering of each merged ensemble with a range of values of *perp* and N and compute the integrated silhouette score (Eq. 7) of the resulting cluster assignments. We identify two locally optimal t-SNE projections for coavalent adduct ensembles ($perp$ = 600, N=4 clusters and $perp$ = 300, N=20 clusters) and two locally optimal t-SNE projections for ensembles from non-covalent binding simulations ($perp$ = 300, N=4 clusters and $perp$ = 200, N=18 clusters) highlighted by red boxes.

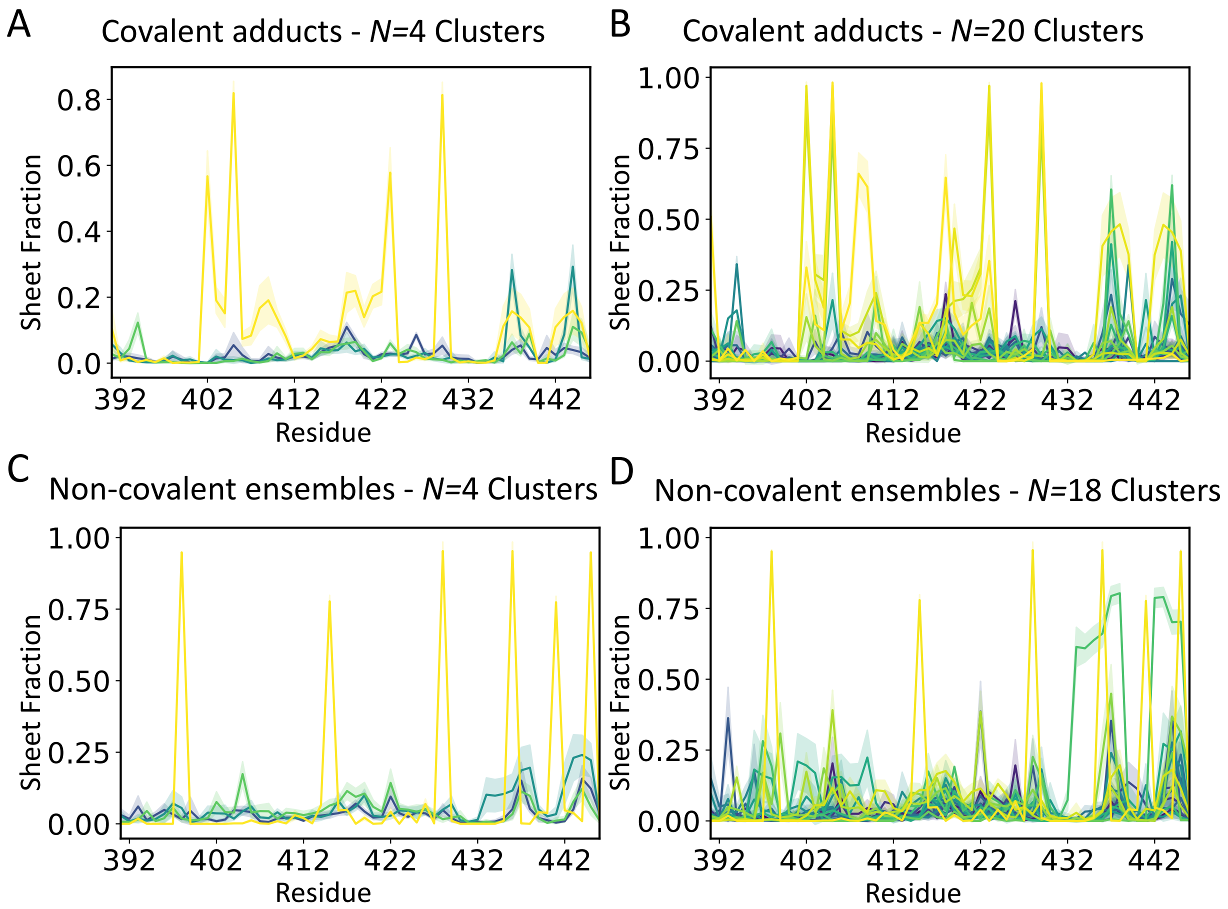

Figure S11: β-sheet propensities of clusters obtained from t-SNE clustering. **A)** β-sheet propensities of covalent adduct clusters identified with $perp$ = 600 and N=4 clusters. **B)** β-sheet propensities of covalent adduct clusters identified with $perp$ = 300 and N=20 clusters. **C)** β-sheet propensities propensities of clusters identified from non-covalent ligand-binding simulations with $perp$ = 300 and N=4 clusters. **D)** β-sheet propensities propensities of clusters identified from non-covalent ligand-binding simulations with $perp$ = 200 and N=18 clusters.

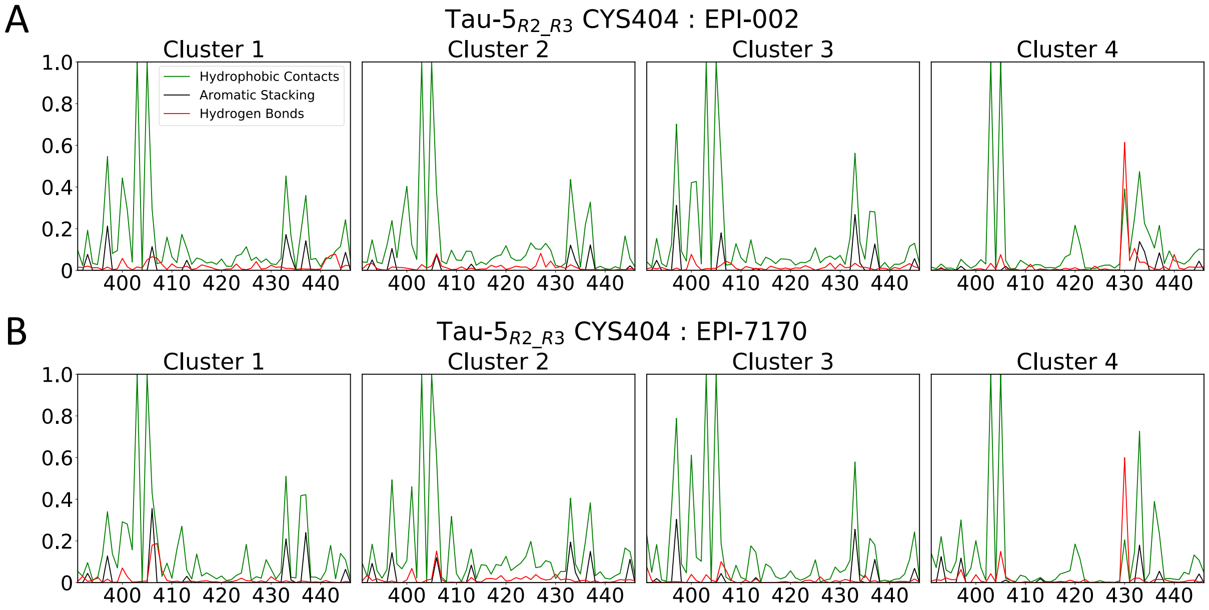

Figure S12: Populations of protein-ligand interactions in Tau-5$_{\mathbf{R2_R3}}$ covalent adduct conformational states identified by t-SNE clustering with N=4 clusters. **A)** Populations of intramolecular interactions between CYS404:EPI-002 and Tau-5$_{\mathrm{R2_R3}}$ residues in each cluster of the Tau-5$_{\mathrm{R2_R3}}$-CYS404:EPI-002 ensemble. **B)** Populations of intramolecular interactions between CYS404:EPI-7170 and Tau-5$_{\mathrm{R2_R3}}$ residues in each cluster of the Tau-5$_{\mathrm{R2_R3}}$-CYS404:EPI-7170 ensemble.

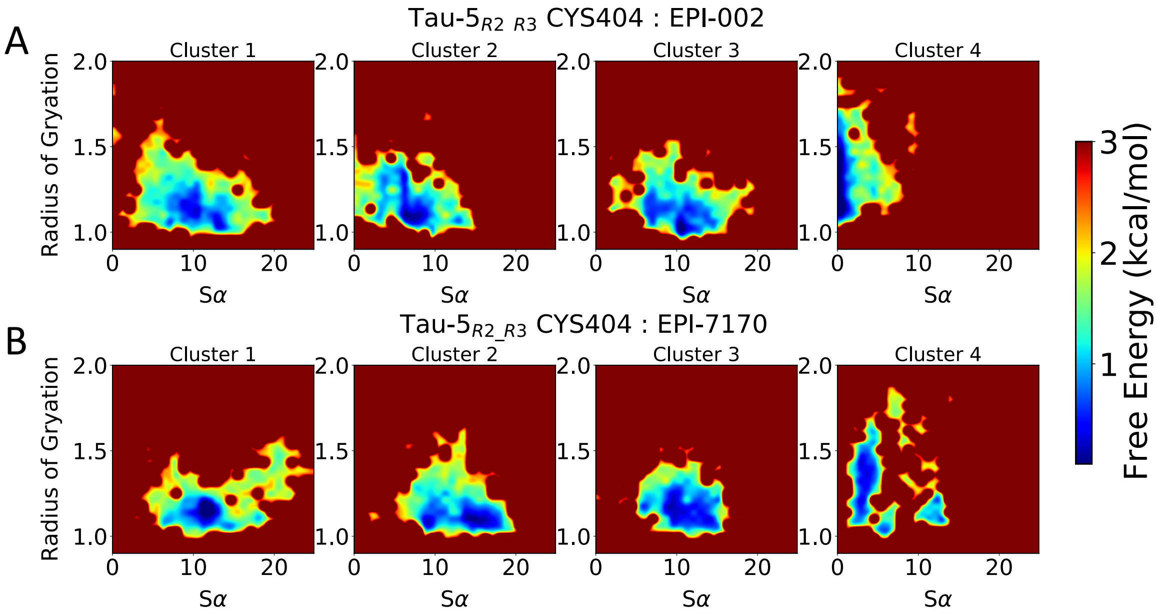

Figure S13: Free energy surfaces of Tau-5$_{\mathbf{R2_R3}}$ covalent adduct conformational states identified by t-SNE clustering with N=4 clusters. Free energy surfaces as a function of the radius of gyration (reported in nm) and Sα of Tau-5$_{\mathrm{R2_R3}}$ conformations for covalent adduct conformational states identified by t-SNE clustering with $perp$ = 600 and N=4 clusters for **A)** Tau-5$_{\mathrm{R2_R3}}$-CYS404:EPI-002 and **B)** Tau-5$_{\mathrm{R2_R3}}$-CYS404:EPI-7170.

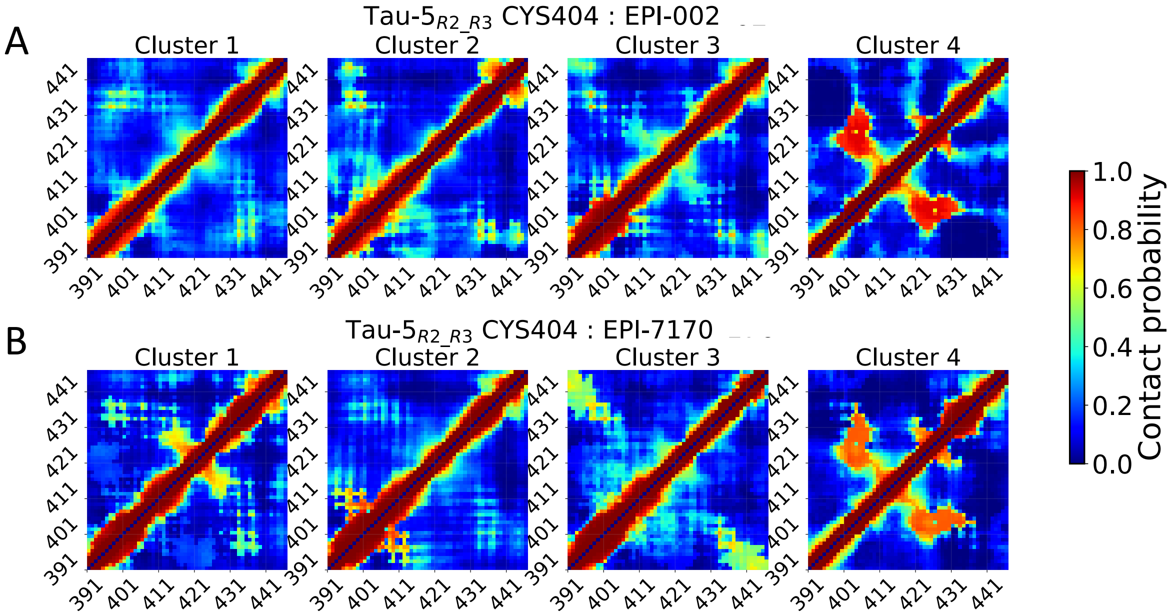

Figure S14: Intramolecular contact populations of Tau-5$_{\mathbf{R2_R3}}$ covalent adduct conformational states identified by t-SNE clustering with N=4 clusters. Intramolecular contact populations of covalent adduct conformational states identified by t-SNE clustering with $perp$ = 600 and N=4 clusters for **A)** Tau-5$_{\mathrm{R2_R3}}$-CYS404:EPI-002 and **B)** Tau-5$_{\mathrm{R2_R3}}$-CYS404:EPI-7170. Contacts between residues are defined using a distance cutoff of 12Å between closest heavy atoms.

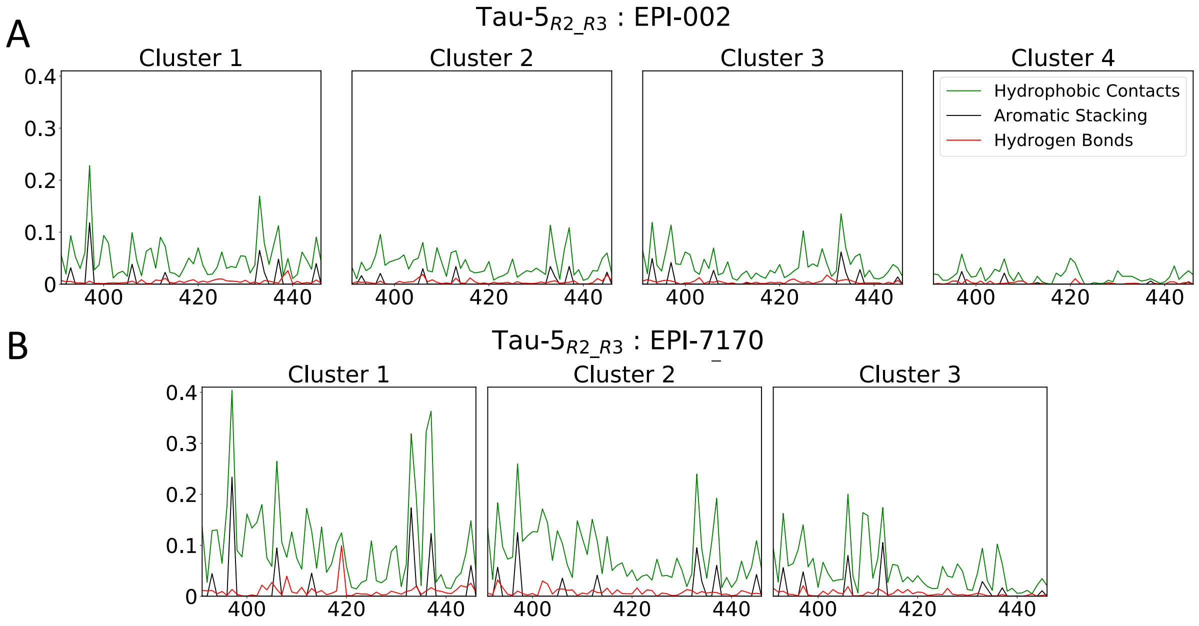

Figure S15: Populations of intermolecular protein-ligand interactions in Tau-5$_{\mathrm{R2_R3}}$ conformational states identified from non-covalent ligand-binding simulations of EPI-002 and EPI-7170 by t-SNE clustering with N=4 clusters. A) Populations of intermolecular interactions between EPI-002 and Tau-5$_{\mathrm{R2_R3}}$ in each cluster. **B)** Populations of intermolecular interactions between EPI-7170 and Tau-5$_{\mathrm{R2_R3}}$ in clusters 1-3. The interaction plot of cluster 4 is excluded because only one frame from was this ensemble was assigned to the this cluster.

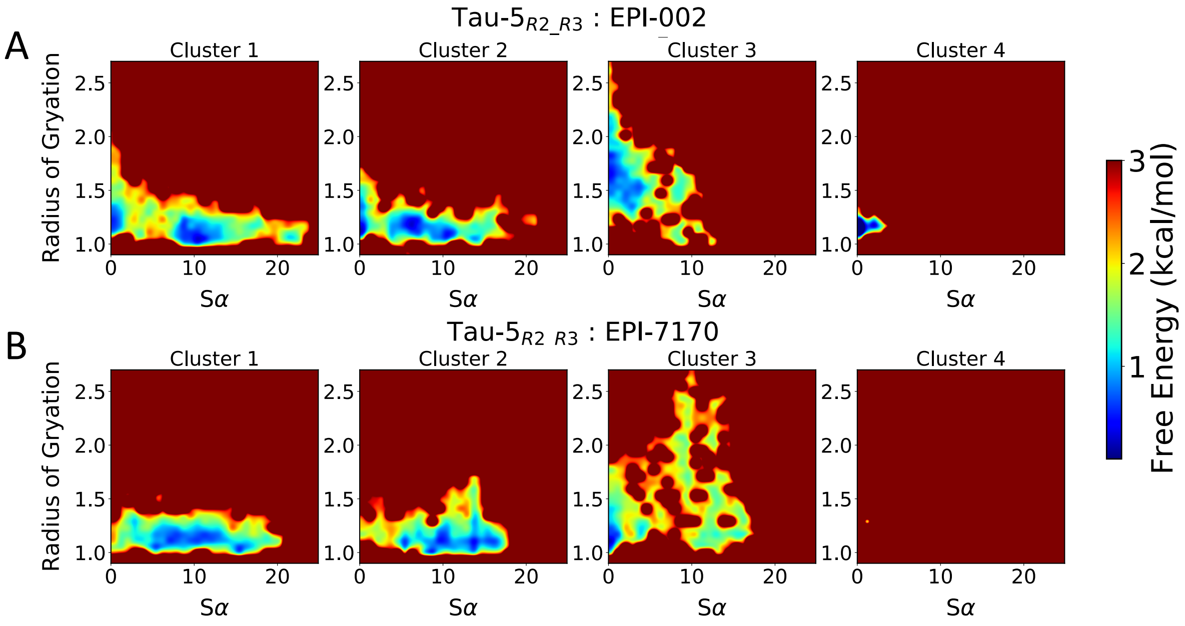

Figure S16: Free energy surfaces of Tau-5$_{\mathbf{R2_R3}}$ conformational states identified from non-covalent ligand binding simulations by t-SNE clustering with $\boldsymbol{N}$=4 clusters. Free energy surfaces as a function of the radius of gyration (reported in nm) and Sα of Tau-5$_{\mathrm{R2_R3}}$ conformations of conformational states identified from non-covalent ligand-binding simulations of EPI-002 and EPI-7170 by t-SNE clustering with *perp* = 300 N=4 clusters for **A)** Tau-5$_{\mathrm{R2_R3}}$ in the presence of EPI-002 and **B)** Tau-5$_{\mathrm{R2_R3}}$ in the presence of EPI-7170.

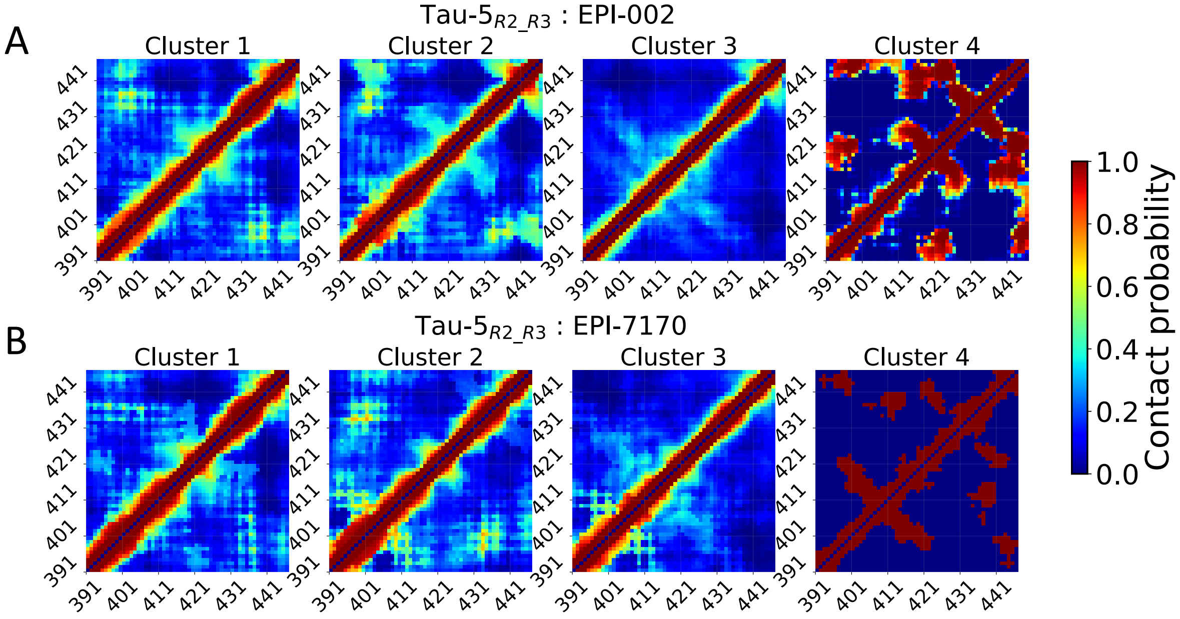

Figure S17: Intramolecular contact populations of Tau-5$_{\mathbf{R2_R3}}$ conformational states identified from non-covalent ligand binding simulations by t-SNE clustering with $\boldsymbol{N}$=4 clusters. Intramolecular contact populations of Tau-5$_{\mathrm{R2_R3}}$ conformational states identified by t-SNE clustering with $perp$ = 300 and N=4 clusters for **A)** Tau-5$_{\mathrm{R2_R3}}$ in the presence of EPI-002 and **B)** Tau-5$_{\mathrm{R2_R3}}$ in the presence of EPI-7170. We note that cluster 4 contains only one frame from this ensemble. Contacts between residues are defined using a distance cutoff of 12Å between closest heavy atoms.

Table S2: Cluster population (p), helical globule population (p_{Glob}) and helix fraction (HF) of clusters obtained from t-SNE clustering of a merged ensemble of Tau-$5_{\mathrm{R2\_R3}}$-CYS404:EPI-002 and Tau-$5_{\mathrm{R2\_R3}}$-CYS404:EPI-7170 conformations with N=20 clusters. We compare the properties of the clusters in the merged ensemble to the properties of the clustered conformations from the the individual Tau-$5_{\mathrm{R2\_R3}}$-CYS404:EPI-002 and Tau-$5_{\mathrm{R2\_R3}}$-CYS404:EPI-7170 ensembles.

Cluster	Merged Ensembles			Tau-$5_{\mathrm{R2\_R3}}$-CYS404:EPI-002			Tau-$5_{\mathrm{R2\_R3}}$-CYS404:EPI-7170		
	p	p_{Glob}	HF	p	p_{Glob}	HF	p	p_{Glob}	HF
1	0.06	0.80	0.42	0.01	0.29	0.20	0.11	0.85	0.44
2	0.07	0.88	0.36	0.04	0.66	0.05	0.10	0.96	0.39
3	0.06	0.82	0.35	0.06	0.76	0.29	0.07	0.87	0.40
4	0.04	0.90	0.35	0.01	0.66	0.27	0.07	0.95	0.37
5	0.05	0.55	0.34	0.05	0.58	0.29	0.06	0.51	0.38
6	0.04	0.75	0.33	0.07	0.75	0.33	0.02	0.72	0.32
7	0.05	0.65	0.04	0.07	0.58	0.29	0.03	0.80	0.32
8	0.06	0.94	0.30	0.08	0.92	0.30	0.04	0.97	0.37
9	0.05	0.92	0.30	0.00	0.67	0.30	0.10	0.93	0.30
10	0.04	0.84	0.30	0.06	0.87	0.30	0.02	0.71	0.29
11	0.05	0.79	0.30	0.07	0.78	0.28	0.03	0.80	0.34
12	0.06	0.59	0.09	0.06	0.38	0.28	0.06	0.81	0.36
13	0.04	0.68	0.29	0.05	0.54	0.26	0.04	0.87	0.34
14	0.07	0.89	0.28	0.10	0.86	0.28	0.04	0.96	0.30
15	0.02	0.25	0.24	0.00	0.00	0.17	0.04	0.26	0.24
16	0.04	0.03	0.22	0.04	0.00	0.09	0.04	0.05	0.28
17	0.04	0.44	0.12	0.01	0.12	0.11	0.07	0.49	0.22
18	0.05	0.04	0.12	0.06	0.08	0.06	0.05	0.00	0.13
19	0.05	0.00	0.06	0.09	0.00	0.06	0.00	0.00	0.10
20	0.04	0.00	0.06	0.07	0.00	0.06	0.00	0.00	0.08

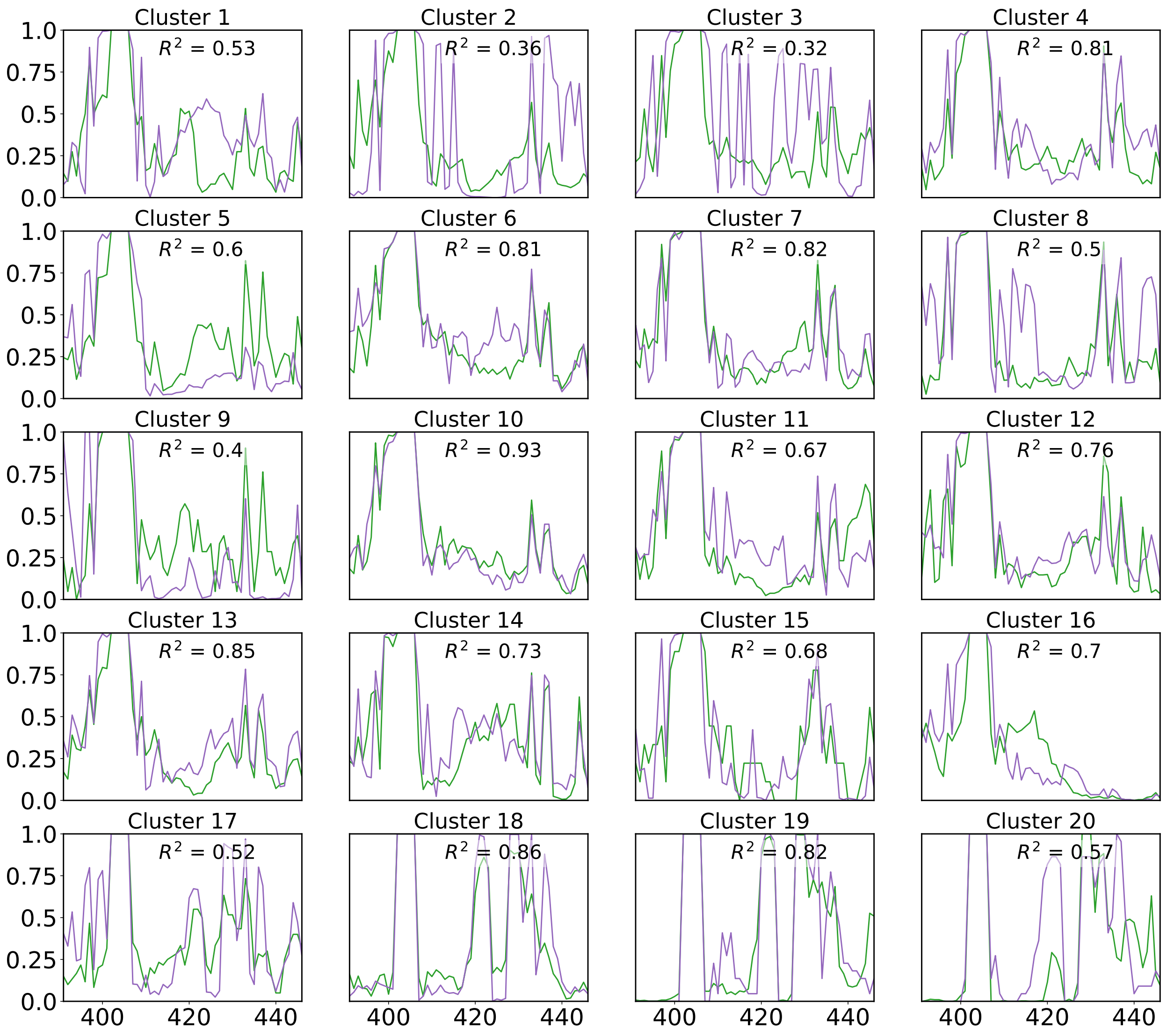

Figure S18: Intramolecular contact probabilities observed between covalently modified CYS404 residues and Tau-5$_{\mathrm{R2_R3}}$ residues in covalent adduct conformational states identified by t-SNE clustering with N=20 clusters. Populations of intramolecular contacts between Tau-5$_{\mathrm{R2_R3}}$ residues and CYS404:EPI-002 (green) and Tau-5$_{\mathrm{R2_R3}}$ residues and CYS404:EPI-7170 (purple) are shown for each cluster identified by t-SNE clustering with *perp*=300 and N=20 clusters. The coefficient of determination (R^2) of the populations of intramolecular contacts formed by CYS404:EPI-002 and CYS404:EPI-7170 are reported for each cluster.

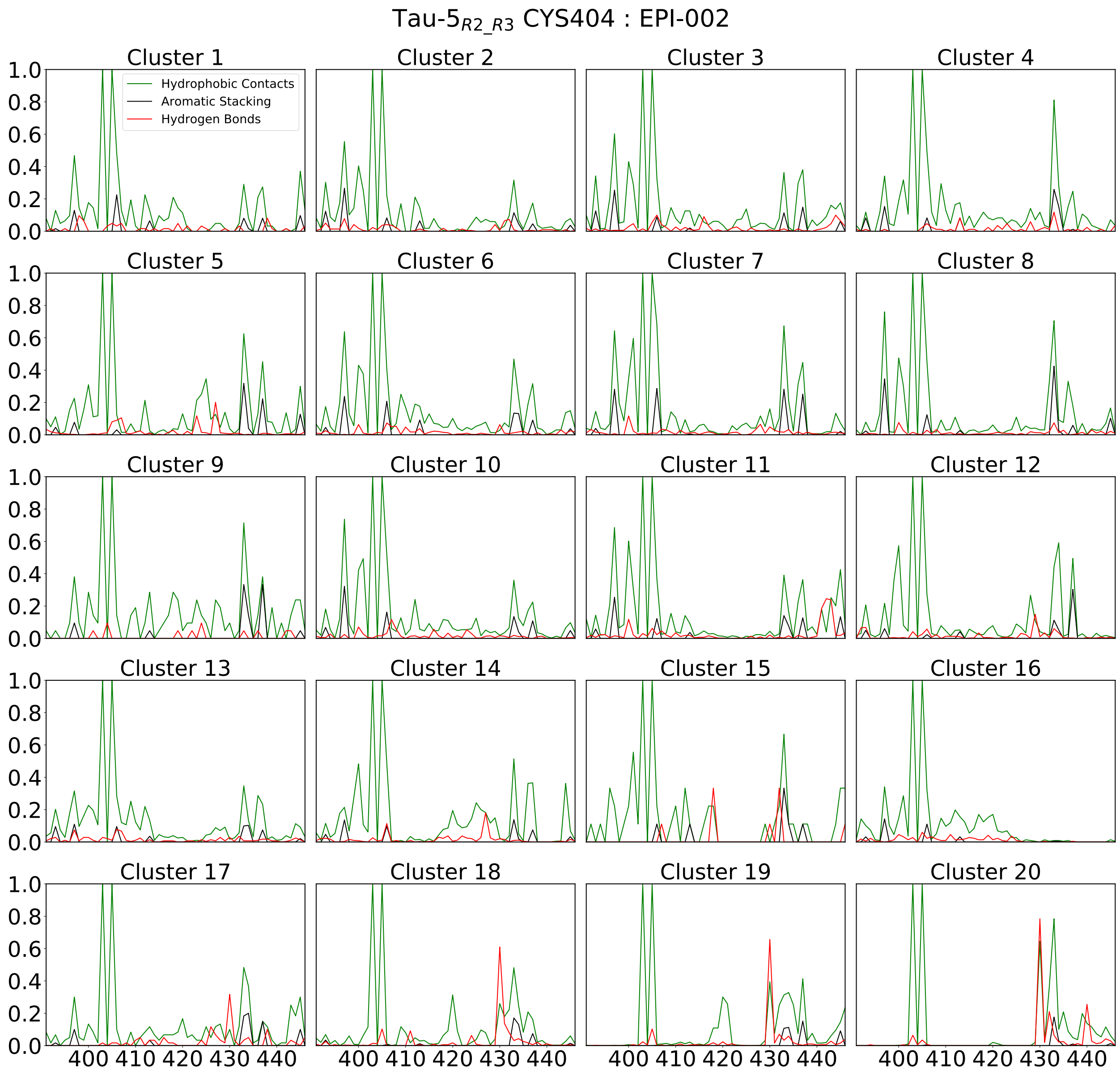

Figure S19: Populations of protein-ligand interactions in Tau-5$_{R2_R3}$-CYS404:EPI-002 covalent adduct conformational states identified by t-SNE clustering with N=20 clusters. Populations of intramolecular interactions between CYS404:EPI-002 and Tau-5$_{R2_R3}$ residues in each cluster of the Tau-5$_{R2_R3}$-CYS404:EPI-002 ensemble.

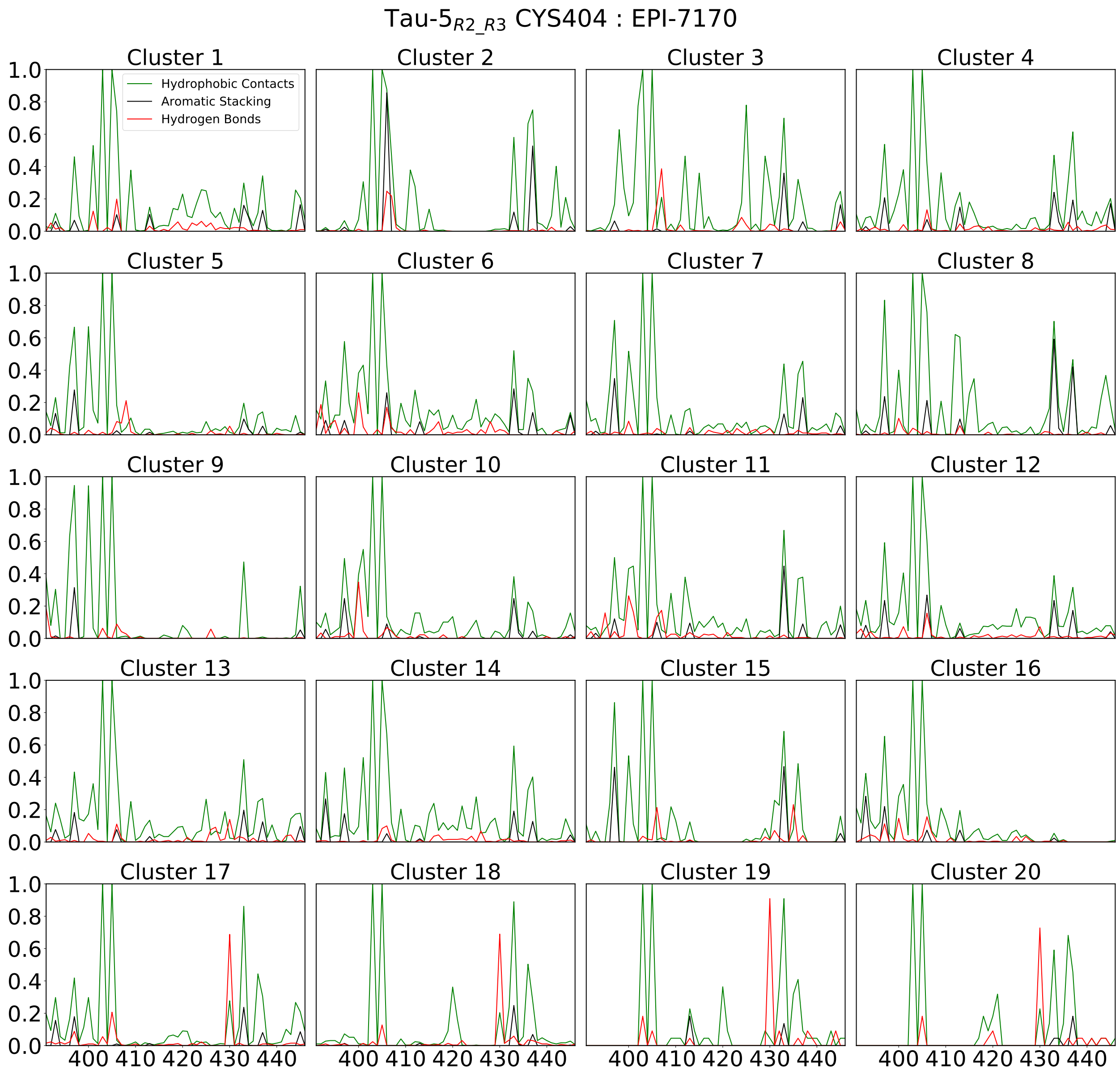

Figure S20: Populations of protein-ligand interactions in Tau-5$_{R2_R3}$-CYS404:EPI-7170 covalent adduct conformational states identified by t-SNE clustering with N=20 clusters. Populations of intramolecular interactions between CYS404:EPI-7170 and Tau-5$_{R2_R3}$ residues in each cluster of the Tau-5$_{R2_R3}$-CYS404:EPI-7170 ensemble.

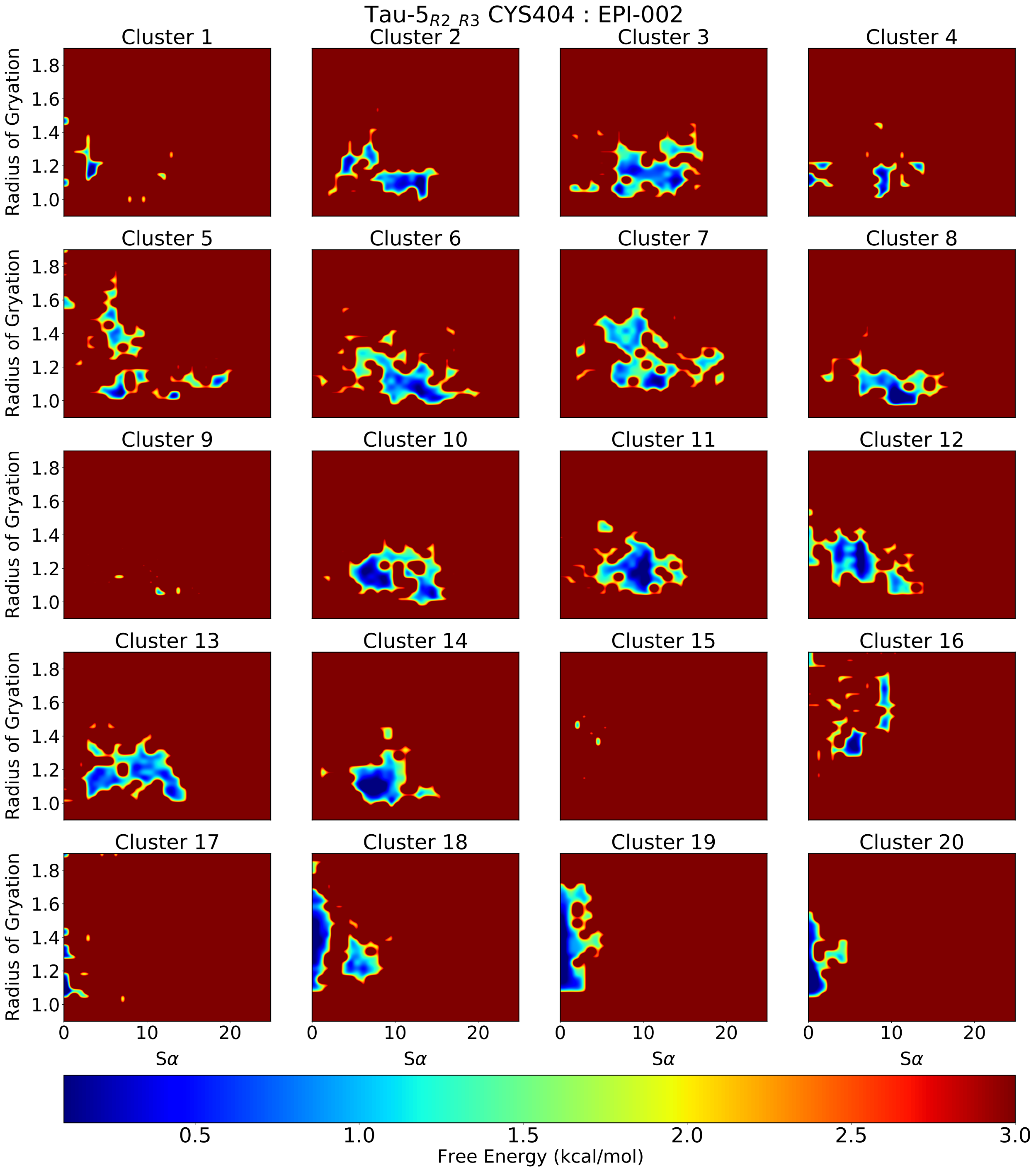

Figure S21: Free energy surfaces of Tau-5$_{\mathbf{R2_R3}}$-CYS404:EPI-002 covalent adduct conformational states identified by t-SNE clustering with N=20 clusters. Free energy surfaces as a function of the radius of gyration (reported in nm) and Sα of Tau-5$_{\mathrm{R2_R3}}$ conformations for Tau-5$_{R2_R3}$-CYS404:EPI-002 covalent adduct conformational states identified by t-SNE clustering with $perp$ = 300 and N=20 clusters.

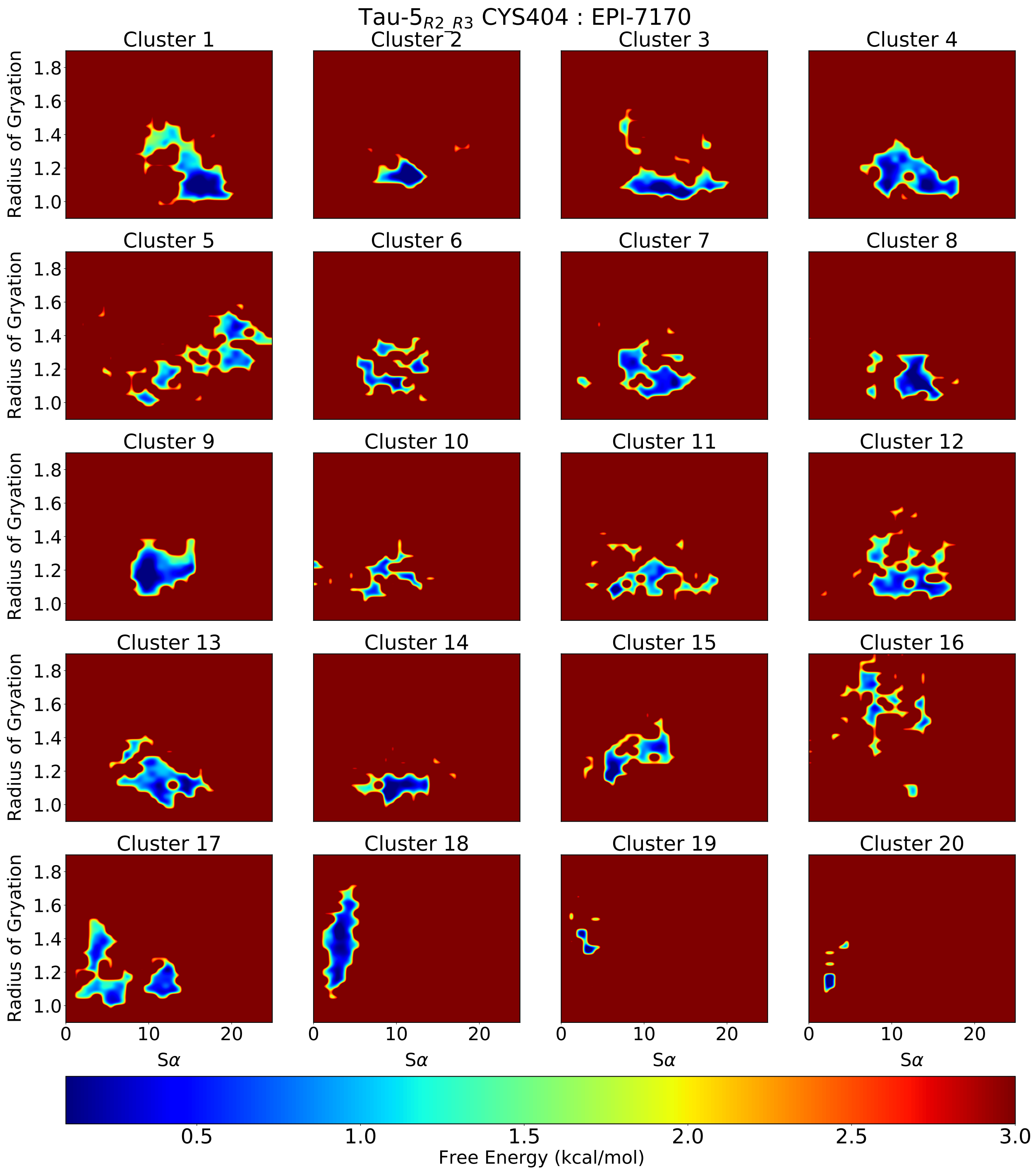

Figure S22: Free energy surfaces of Tau-5$_{\mathbf{R2_R3}}$-CYS404:EPI-7170 covalent adduct conformational states identified by t-SNE clustering with N=20 clusters. Free energy surfaces as a function of the radius of gyration (reported in nm) and Sα of Tau-5$_{\mathrm{R2_R3}}$ conformations for Tau-5$_{R2_R3}$-CYS404:EPI-7170 covalent adduct conformational states identified by t-SNE clustering with $perp$ = 300 and N=20 clusters.

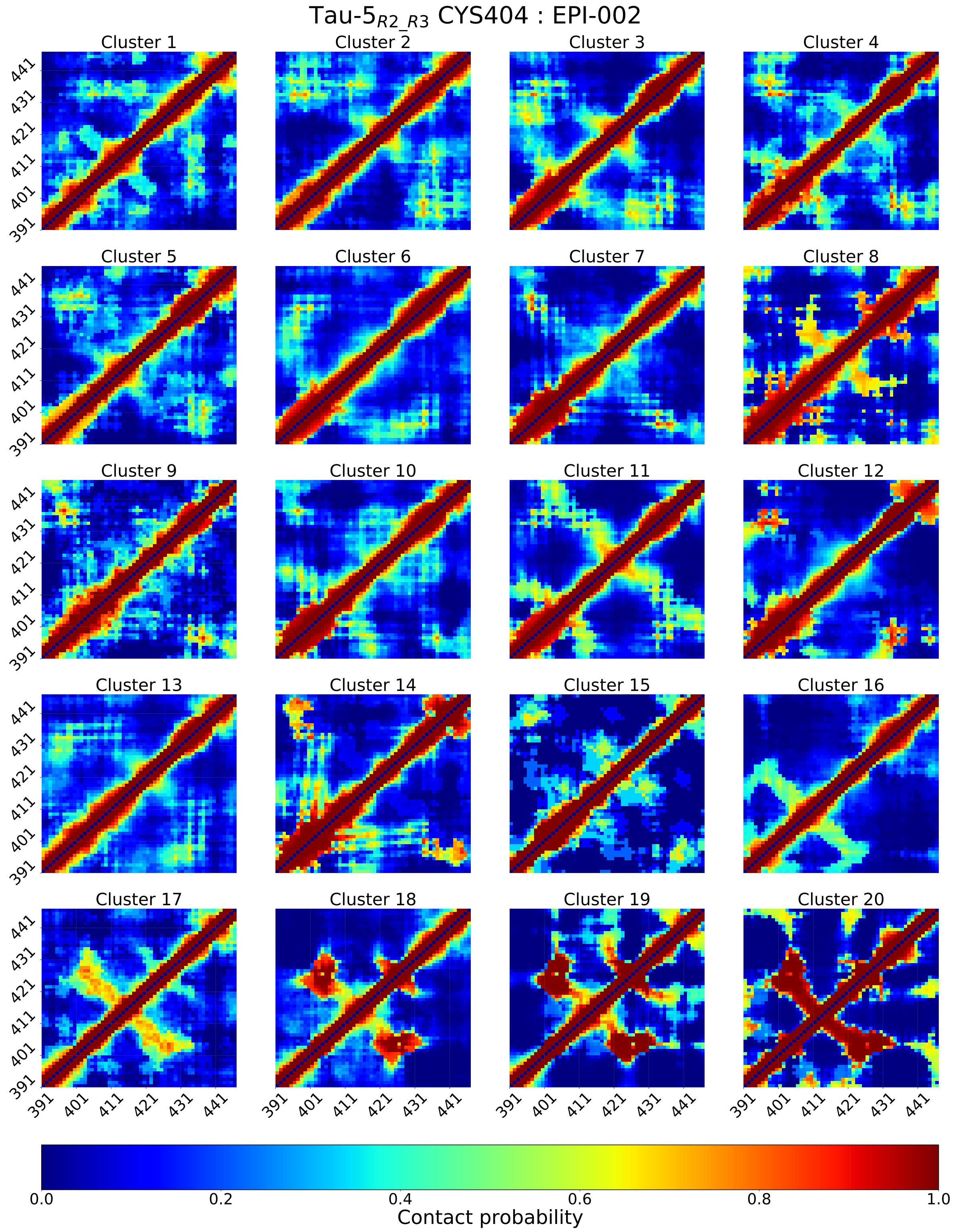

Figure S23: Intramolecular contact populations of Tau-5$_{\mathbf{R2_R3}}$-CYS404:EPI-002 covalent adduct conformational states identified by t-SNE clustering with N=20 clusters. Intramolecular contact populations of Tau-5$_{\mathrm{R2_R3}}$-CYS404:EPI-002 covalent adduct conformational states identified by t-SNE clustering with $perp$ = 300 and N=20 clusters. Contacts between residues are defined using a distance cutoff of 12Å between closest heavy atoms.

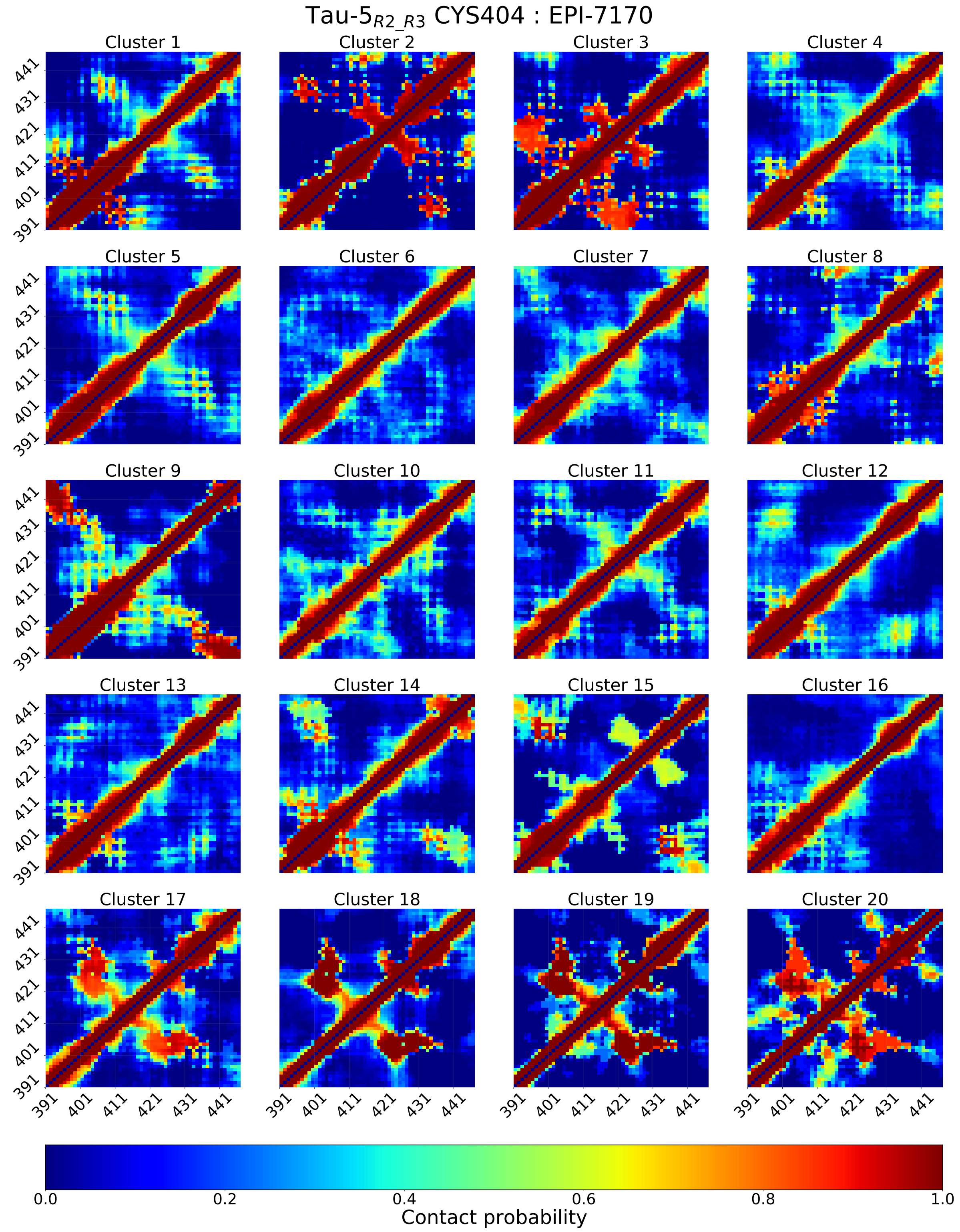

Figure S24: Intramolecular contact populations of Tau-5$_{\mathbf{R2_R3}}$-CYS404:EPI-7170 covalent adduct conformational states identified by t-SNE clustering with N=20 clusters. Intramolecular contact populations of Tau-5$_{\mathrm{R2_R3}}$-CYS404:EPI-7170 covalent adduct conformational states identified by t-SNE clustering with $perp$ = 300 and N=20 clusters. Contacts between residues are defined using a distance cutoff of 12Å between closest heavy atoms.

Table S3: Cluster population (p), bound fraction (BF), helical globule population (p_{Glob}) and helix fraction (HF) of clusters obtained from t-SNE clustering of a merged ensemble containing all frames from Tau-5_{R2_R3}:EPI-002 and Tau-5_{R2_R3}:EPI-7170 non-covalent binding simulations with N=18 clusters. We compare the properties of the clusters in the merged ensemble to the properties of the clustered conformations from the individual Tau-5_{R2_R3}:EPI-002 and Tau-5_{R2_R3}:EPI-7170 non-covalent binding simulations.

Cluster	Merged Ensembles				Tau-5_{R2_R3}:EPI-002				Tau-5_{R2_R3}:EPI-7170			
	p	BF	p_{Glob}	HF	p	BF	p_{Glob}	HF	p	BF	p_{Glob}	HF
1	0.08	0.74	0.89	0.38	0.07	0.67	0.90	0.36	0.09	0.80	0.88	0.40
2	0.05	0.66	0.78	0.36	0.04	0.60	0.70	0.35	0.06	0.70	0.82	0.37
3	0.07	0.68	0.90	0.35	0.04	0.57	0.81	0.32	0.10	0.72	0.93	0.37
4	0.06	0.60	0.68	0.32	0.04	0.69	0.78	0.38	0.07	0.55	0.63	0.29
5	0.04	0.53	0.89	0.32	0.07	0.50	0.95	0.31	0.02	0.65	0.71	0.34
6	0.07	0.66	0.61	0.31	0.02	0.33	0.62	0.25	0.11	0.73	0.61	0.32
7	0.06	0.53	0.69	0.29	0.08	0.41	0.70	0.32	0.05	0.70	0.68	0.26
8	0.06	0.59	0.70	0.28	0.06	0.39	0.47	0.23	0.07	0.75	0.88	0.32
9	0.05	0.47	0.69	0.27	0.09	0.40	0.64	0.25	0.02	0.76	0.89	0.34
10	0.05	0.68	0.18	0.26	0.02	0.62	0.22	0.16	0.07	0.69	0.17	0.28
11	0.05	0.65	0.66	0.25	0.03	0.45	0.29	0.16	0.06	0.73	0.80	0.28
12	0.05	0.48	0.00	0.25	0.03	0.36	0.00	0.12	0.08	0.51	0.00	0.28
13	0.06	0.26	0.44	0.23	0.11	0.21	0.43	0.22	0.01	0.75	0.56	0.27
14	0.03	0.71	0.71	0.22	0.00	0.65	0.61	0.17	0.05	0.72	0.72	0.22
15	0.05	0.56	0.19	0.19	0.06	0.37	0.07	0.16	0.05	0.76	0.14	0.27
16	0.05	0.52	0.09	0.15	0.05	0.57	0.03	0.12	0.05	0.49	0.14	0.14
17	0.05	0.49	0.08	0.14	0.06	0.47	0.05	0.08	0.04	0.51	0.13	0.16
18	0.06	0.30	0.00	0.03	0.13	0.30	0.00	0.03	-	-	-	-

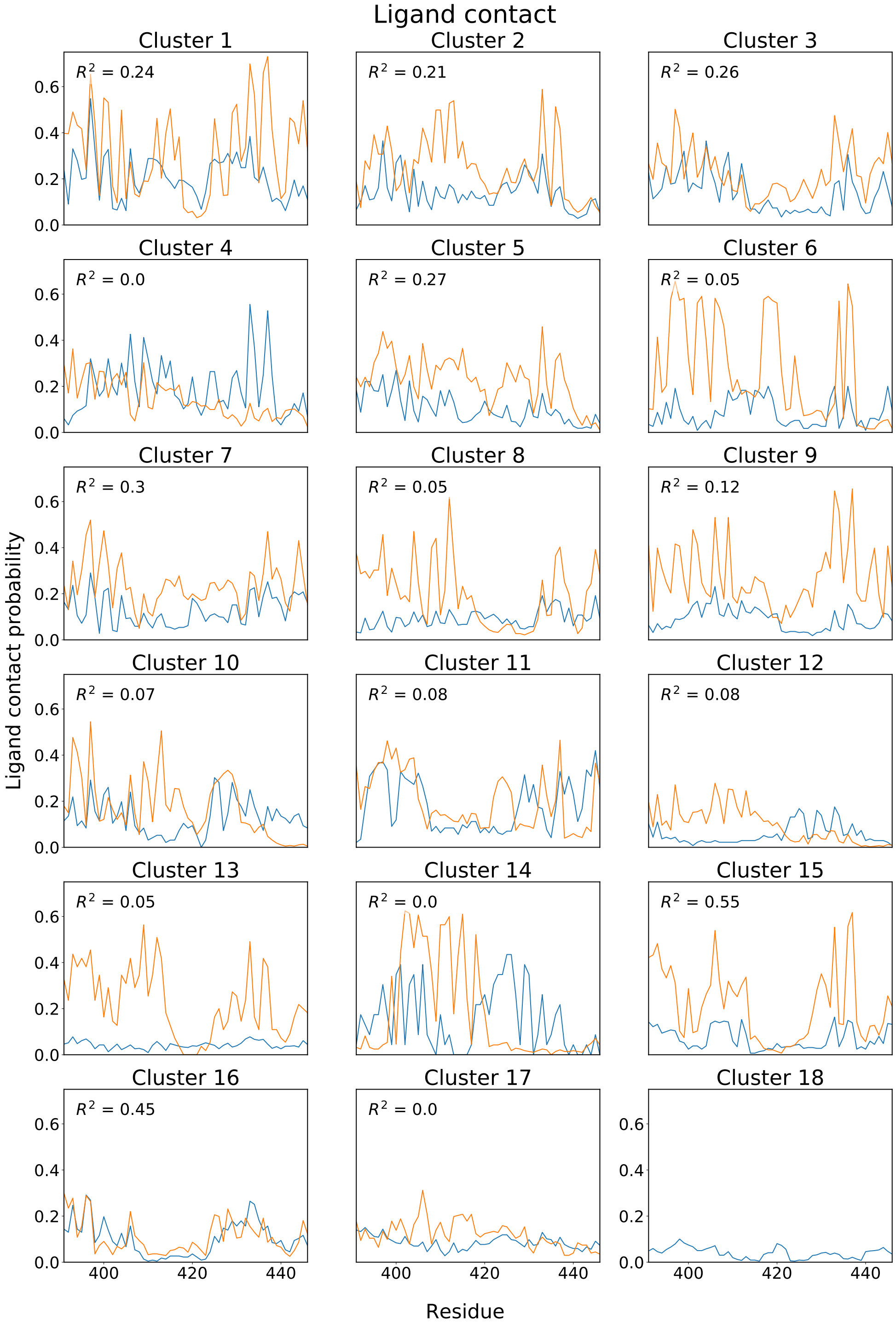

Figure S25: Populations of intermolecular protein-ligand contacts in Tau-5$_{\mathrm{R2_R3}}$ conformational states identified by t-SNE clustering of non-covalent ligand binding simulations of EPI-002 and EPI-7170 with N=18 clusters. Populations of intermolecular contacts between Tau-5$_{\mathrm{R2_R3}}$ and EPI-002 (blue) and Tau-5$_{\mathrm{R2_R3}}$ and EPI-7170 (orange) are shown for each cluster identified by t-SNE clustering with $perp$=200 and N=18 clusters. The coefficient of determination (R^2) of the populations of intramolecular contacts formed between Tau-5$_{\mathrm{R2_R3}}$ and EPI-002 and Tau-5$_{\mathrm{R2_R3}}$ and EPI-7170 are reported for each cluster.

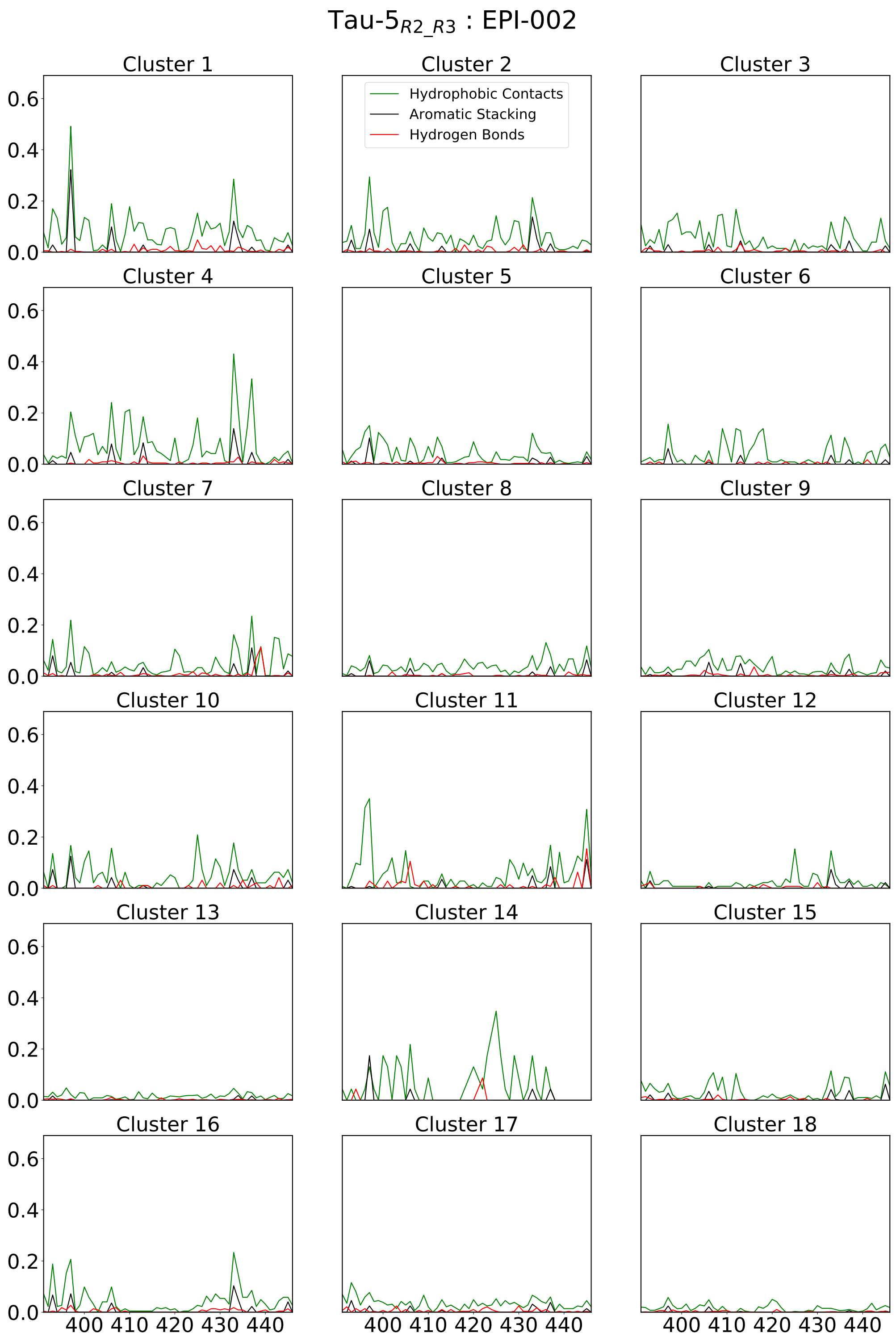

Figure S26: Populations of Tau-5$_{R2_R3}$:EPI-002 intermolecular protein-ligand interactions in Tau-5$_{R2_R3}$ conformational states identified by t-SNE clustering non-covalent ligand binding simulations of EPI-002 and EPI-7170 with N=18 clusters. Populations of intermolecular interactions between EPI-002 and Tau-5$_{R2_R3}$ in each cluster.

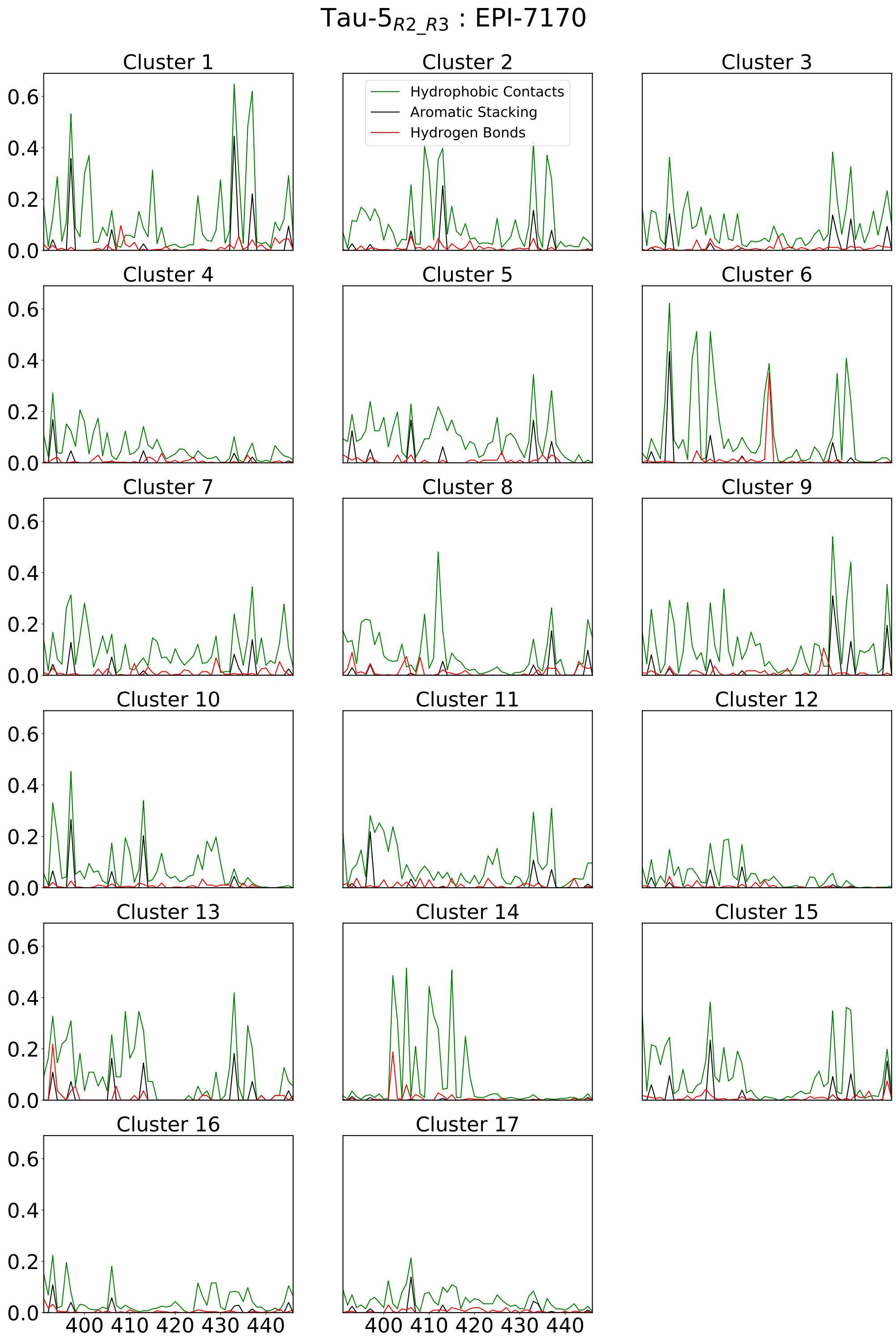

Figure S27: Populations of Tau-5$_{\mathbf{R2_R3}}$:EPI-7170 intermolecular protein-ligand interactions in Tau-5$_{\mathbf{R2_R3}}$ conformational states identified by t-SNE clustering non-covalent ligand binding simulations of EPI-002 and EPI-7170 with N=18 clusters. Populations of intermolecular interactions between EPI-7170 and Tau-5$_{\mathrm{R2_R3}}$ in each cluster. The interaction plot of cluster 18 is excluded because only one frame from this ensemble was assigned to the this cluster.

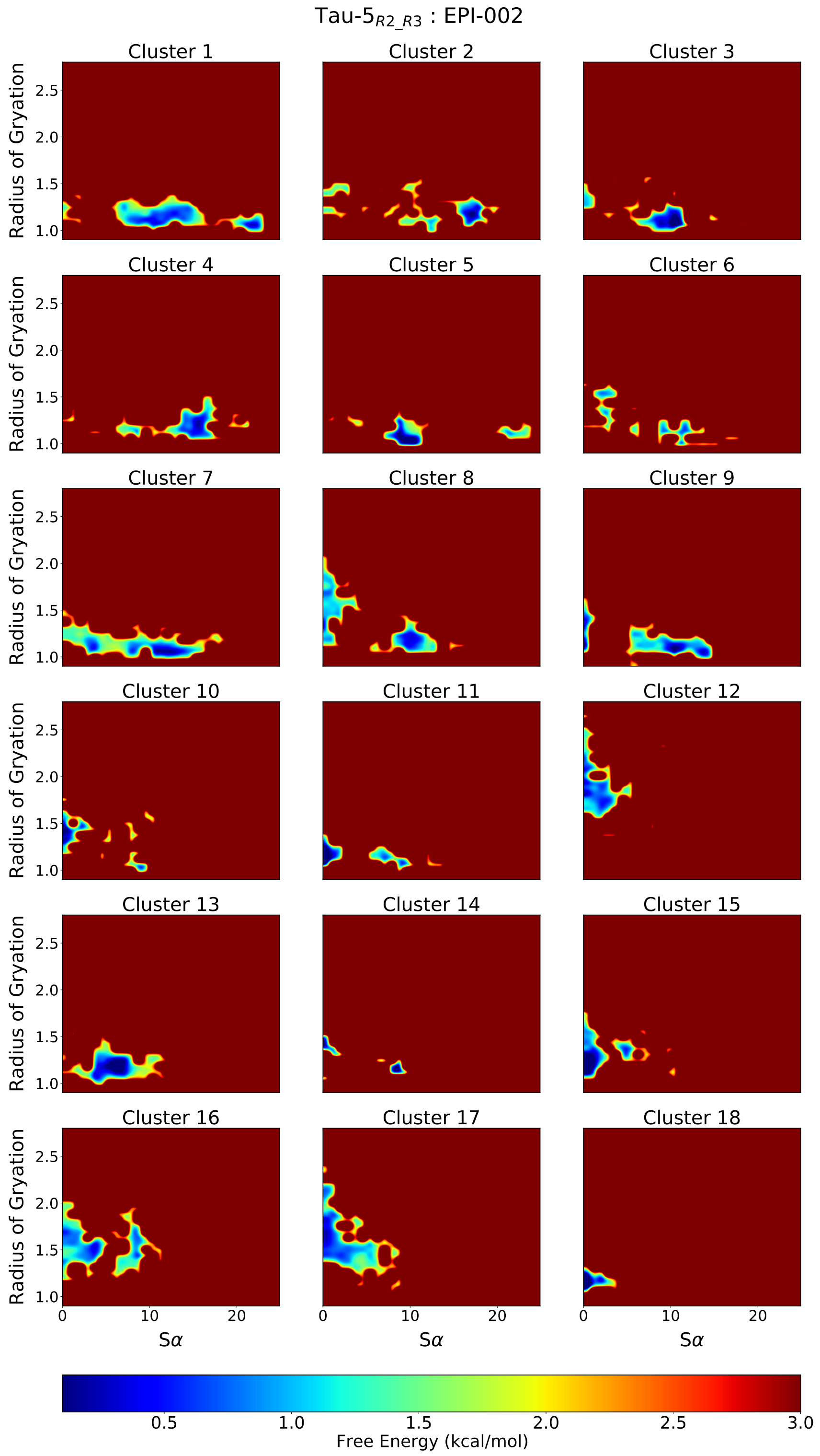

Figure S28: Free energy surfaces of conformational states from a non-covalent ligand binding simulation of Tau-5$_{\mathrm{R2_R3}}$ and EPI-002 identified by t-SNE clustering with N=18 clusters. Free energy surfaces as a function of the radius of gyration (reported in nm) and Sα of Tau-5$_{\mathrm{R2_R3}}$ conformations of conformational states from a non-covalent ligand-binding simulation of Tau-5$_{\mathrm{R2_R3}}$ in the presence of EPI-002 identified by t-SNE clustering with $perp$ = 200 and N=18 clusters.

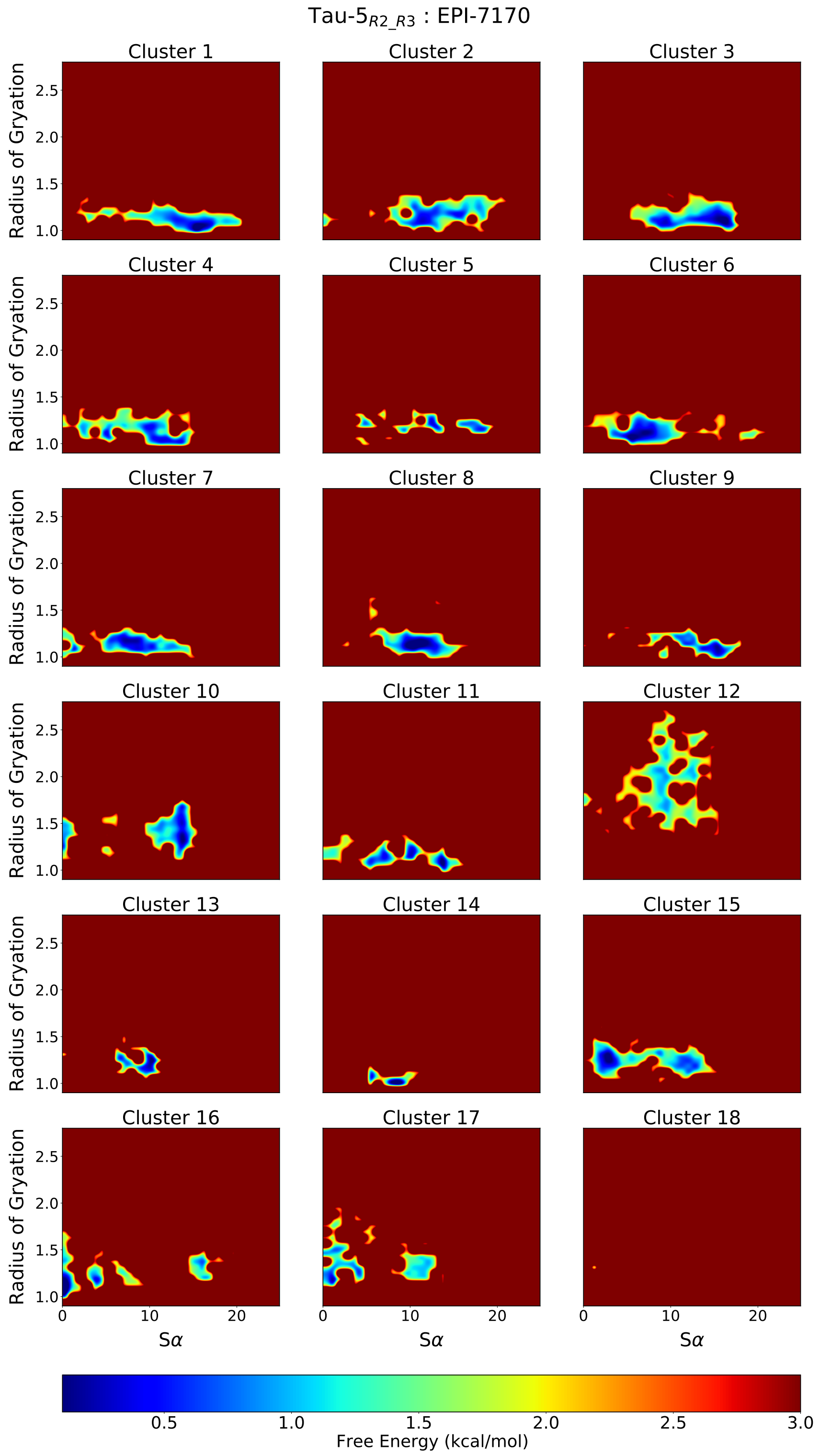

Figure S29: Free energy surfaces of conformational states from a non-covalent ligand binding simulation of Tau-5$_{\mathbf{R2_R3}}$ and EPI-7170 identified by t-SNE clustering with N=18 clusters. Free energy surfaces as a function of the radius of gyration (reported in nm) and Sα of Tau-5$_{\mathrm{R2_R3}}$ conformations of conformational states from a non-covalent ligand-binding simulation of Tau-5$_{\mathrm{R2_R3}}$ in the presence of EPI-7170 identified by t-SNE clustering with $perp$ = 200 and N=18 clusters.

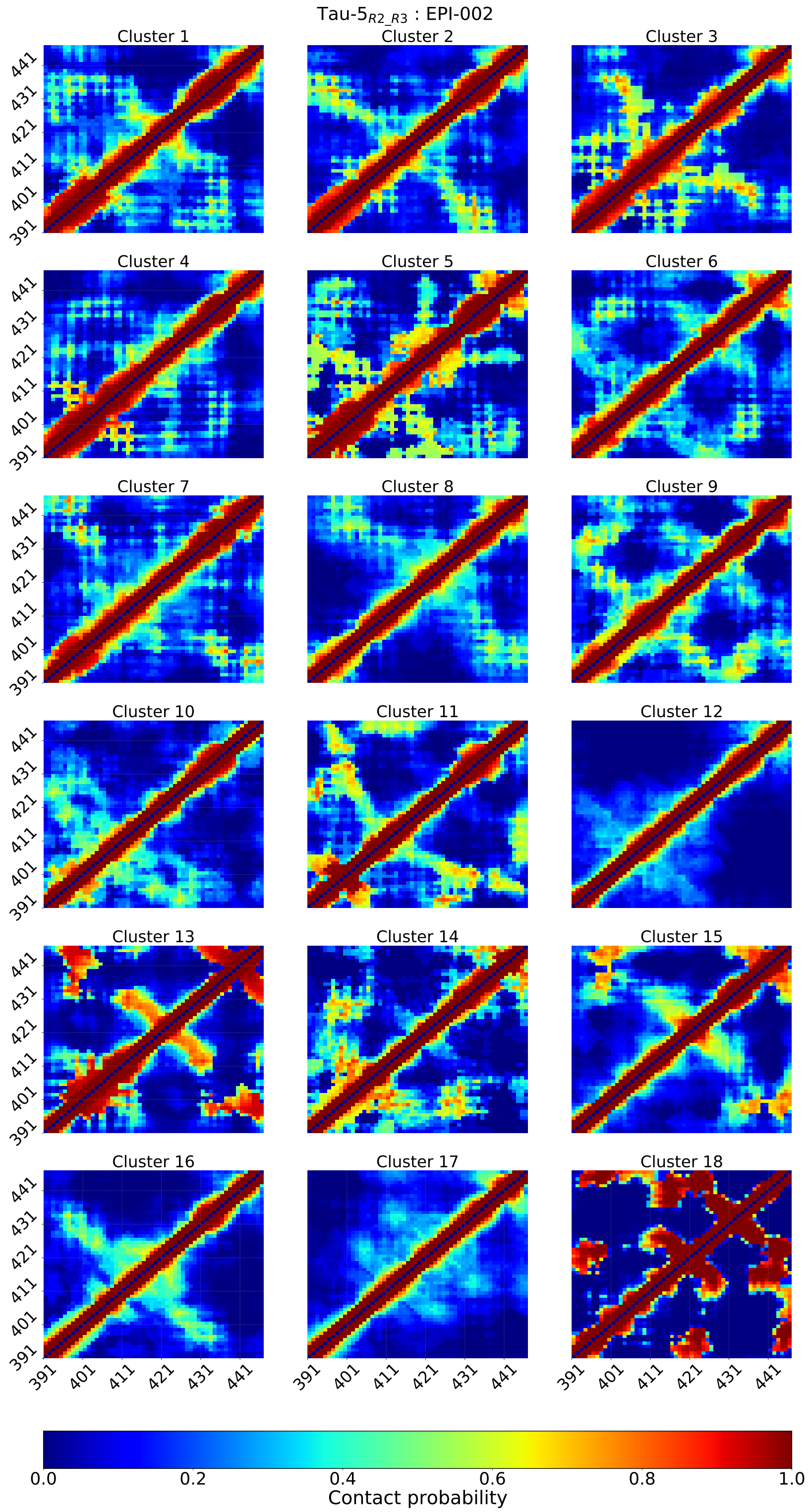

Figure S30: Intramolecular contact populations of Tau-5$_{\mathbf{R2_R3}}$ conformational states identified by t-SNE clustering of a non-covalent ligand-binding simulation of EPI-002 with N=18 clusters. Intramolecular contact populations of Tau-5$_{\mathrm{R2_R3}}$ conformational states identified from a non-covalent EPI-002 binding simulation by t-SNE clustering with $perp$ = 200 and N=18 clusters. We note only one frame from this ensemble was assigned to cluster 18. Contacts between residues are defined using a distance cutoff of 12Å between closest heavy atoms.

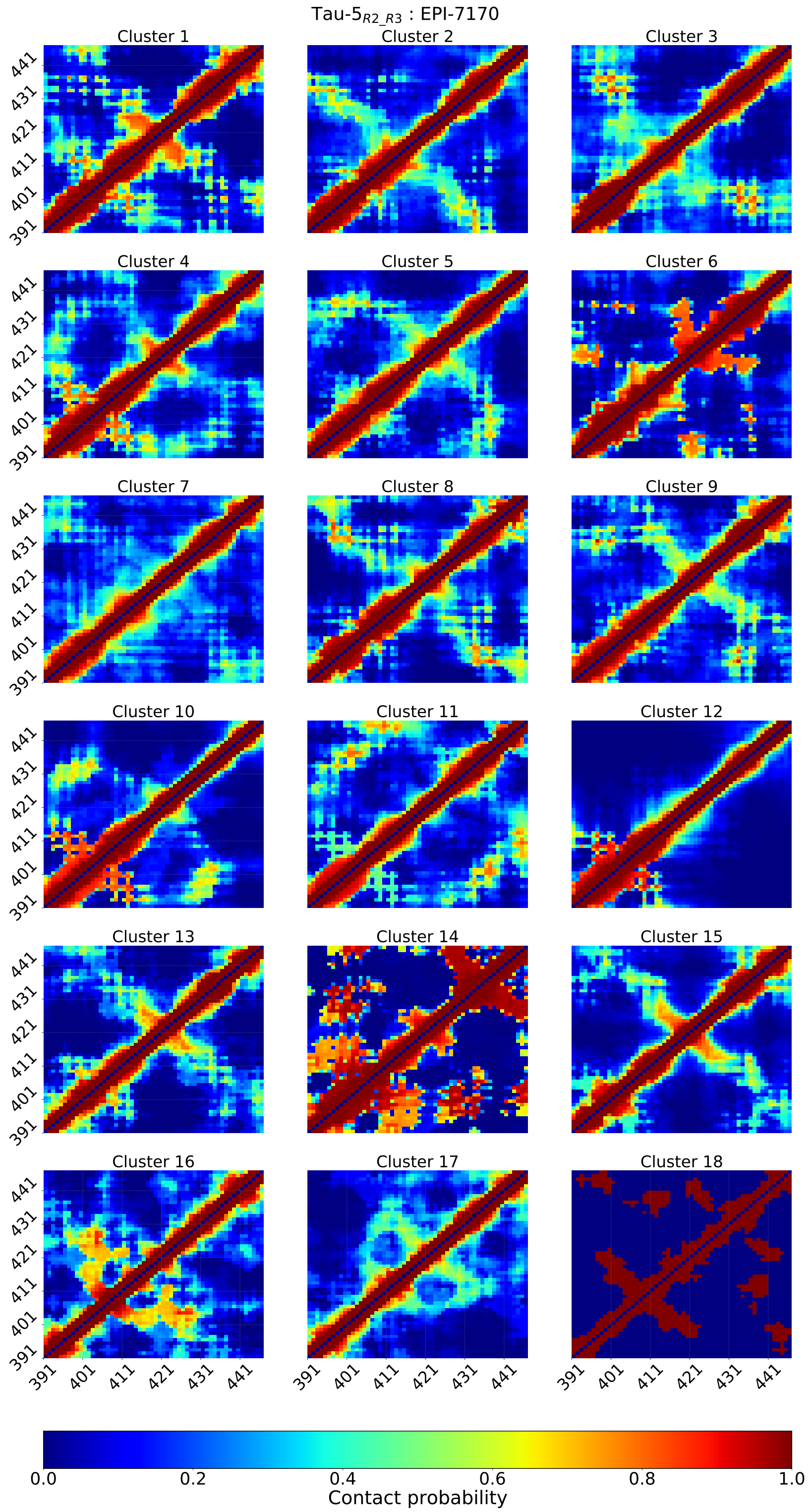

Figure S31: Intramolecular contact populations of Tau-5$_{\mathbf{R2_R3}}$ conformational states identified by t-SNE clustering of a non-covalent ligand-binding simulation of EPI-7170 with $\boldsymbol{N}$=18 clusters. Intramolecular contact populations of Tau-5$_{\mathrm{R2_R3}}$ conformational states identified from a non-covalent EPI-7170 binding simulation by t-SNE clustering with $perp$ = 200 and N=18 clusters. Contacts between residues are defined using a distance cutoff of 12Å between closest heavy atoms.

CYS:EPI-002 (CYE2)

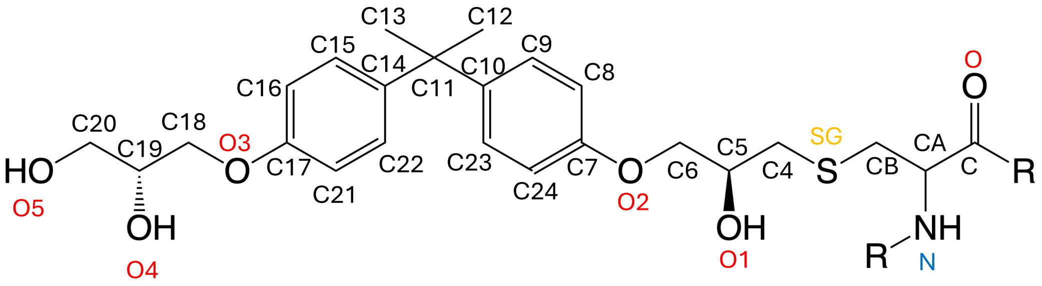

CYS:EPI-7170 (CYE7)

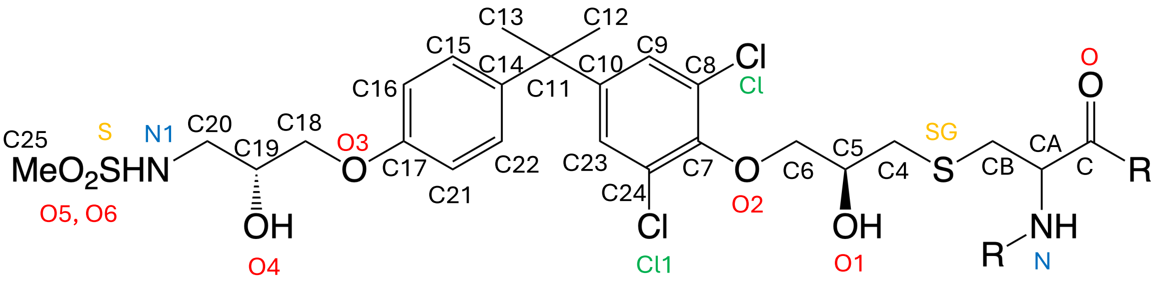

Figure S32: Atom name definitions for CYS:EPI-002 (CYE2) and CYS:EPI-7170 (CYE7) covalently modified cysteine residue force field parameters. Atom names correspond to the force field parameters described in SI tables S4-S10.

Table S4: Partial charge force field parameters for the modified cysteine residue CYS:EPI-002 (CYE2). Atom names, atom types, atom numbers, partial charges and bond list for atoms contained in the modified cysteine residue CYS:EPI-002 (CYE2).

[CYE2]											
[atoms]								[bonds]			
N	N	-0.59637	1	O5	OH	-0.59230	34	-C	N	C12	H17
H	H	0.34653	2	C21	CA	-0.17795	35	N	H	C12	H18
CA	CT	0.05603	3	C22	CA	-0.09146	36	N	CA	C13	H19
HA	H1	0.09526	4	C23	CA	-0.09245	37	CA	CB	C13	H20
CB	CT	-0.03211	5	C24	CA	-0.17795	38	CA	HA	C13	H21
HB1	H1	0.09978	6	H9	H1	0.10431	39	CA	C	C14	C15
HB2	H1	0.09978	7	H10	H1	0.10431	40	CB	SG	C14	C22
SG	S	-0.29345	8	H11	HO	0.42146	41	CB	HB1	C15	C16
C	C	0.64386	9	H12	H1	0.06659	42	CB	HB2	C15	H22
O	O	-0.64528	10	H13	H1	0.06659	43	SG	C4	C16	C17
C4	CT	-0.02315	11	H14	HA	0.14786	44	C4	C5	C16	H23
C5	CT	0.14896	12	H15	HA	0.13831	45	C4	H9	C17	O3
H1	H1	0.06407	13	H16	HC	0.04027	46	C4	H10	C17	C21
O1	OH	-0.60025	14	H17	HC	0.04027	47	C5	O1	O3	C18
C6	CT	0.08390	15	H18	HC	0.04027	48	C5	C6	C18	C19
O2	OS	-0.34685	16	H19	HC	0.04027	49	C5	H1	C18	H24
C7	CA	0.12483	17	H20	HC	0.04027	50	O1	H11	C18	H25
C8	CA	-0.17795	18	H21	HC	0.04027	51	C6	O2	C19	O4
C9	CA	-0.09245	19	H22	HA	0.13931	52	C6	H12	C19	C20
C10	CA	-0.10568	20	H23	HA	0.14334	53	C6	H13	C19	H2
C11	CT	0.04989	21	H24	H1	0.05955	54	O2	C7	O4	H26
C12	CT	-0.08559	22	H25	H1	0.05955	55	C7	C8	C20	O5
C13	CT	-0.08559	23	H26	HO	0.42347	56	C7	C24	C20	H27
C14	CA	-0.10170	24	H27	H1	0.06407	57	C8	C9	C20	H28
C15	CA	-0.09146	25	H28	H1	0.06407	58	C8	H14	O5	H29
C16	CA	-0.17795	26	H29	HO	0.40336	59	C9	C10	C21	C22
C17	CA	0.12483	27	H30	HA	0.14334	60	C9	H15	C21	H30
O3	OS	-0.34486	28	H31	HA	0.13931	61	C10	C11	C22	H31
C18	CT	0.09093	29	H32	HA	0.13831	62	C10	C23	C23	C24
C19	CT	0.10672	30	H33	HA	0.14786	63	C11	C12	C23	H32
H2	H1	0.04798	31					C11	C13	C24	H33
O4	OH	-0.59528	32					C11	C14	C	O
C20	CT	0.12815	33					C12	H16		

Table S5: Dihedral force field parameters of the modified cysteine residue CYS:EPI-002 (CYE2). CYE2 dihedral parameters are applied using the improper dihedral section of the GROMACS CYE2 amino acid template.

[impropers]						
-C	N	CA	C	90.0	1.67360	2
N	CA	C	+N	160.888	2.71960	1
N	CA	C	+N	90.0	-1.0669	1
N	CA	C	+N	90.0	0.3138	2
N	CA	C	+N	90.0	0.2385	3
N	CA	C	+N	90.0	0.1046	4
N	CA	C	+N	90.0	-0.0460	5
-C	CA	N	H			
CA	+N	C	O			
C7	C9	C8	H14	180.00	4.60240	2
C7	C23	C24	H33	180.00	4.60240	2
C8	C10	C9	H15	180.00	4.60240	2
C8	C24	C7	O2	180.00	4.60240	2
C9	C23	C10	C11	180.00	4.60240	2
C10	C24	C23	H32	180.00	4.60240	2
C14	C16	C15	H22	180.00	4.60240	2
C14	C21	C22	H31	180.00	4.60240	2
C15	C17	C16	H23	180.00	4.60240	2
C15	C22	C14	C11	180.00	4.60240	2
C16	C21	C17	O3	180.00	4.60240	2
C17	C22	C21	H30	180.00	4.60240	2

Table S6: Partial charge force field parameters for the modified cysteine residue CYS:EPI-7170 (CYE7). Atom names, atom types, atom numbers, partial charges and bond list for atoms contained in the modified cysteine residue CYS:EPI-7170 (CYE7).

[CYE7]											
[atoms]								[bonds]			
N	N	-0.59637	1	C24	CA	0.03303	37	-C	N	C13	H20
H	H	0.34653	2	H9	H1	0.10431	38	N	H	C13	H21
CA	CT	0.05603	3	H10	H1	0.10431	39	N	CA	C14	C15
HA	H1	0.09526	4	H11	HO	0.42146	40	CA	CB	C14	C22
CB	CT	-0.03211	5	H12	H1	0.06659	41	CA	HA	C15	C16
HB1	H1	0.09978	6	H13	H1	0.06659	42	CA	C	C15	H22
HB2	H1	0.09978	7	CL1	Cl	-0.05300	43	CB	SG	C16	C17
SG	S	-0.29345	8	H15	HA	0.13831	44	CB	HB1	C16	H23
C	C	0.64386	9	H16	HC	0.04027	45	CB	HB2	C17	O3
O	O	-0.64528	10	H17	HC	0.04027	46	SG	C4	C17	C21
C4	CT	-0.02315	11	H18	HC	0.04027	47	C4	C5	O3	C18
C5	CT	0.14896	12	H19	HC	0.04027	48	C4	H9	C18	C19
H1	H1	0.06407	13	H20	HC	0.04027	49	C4	H10	C18	H24
O1	OH	-0.60025	14	H21	HC	0.04027	50	C5	O1	C18	H25
C6	CT	0.08390	15	H22	HA	0.13931	51	C5	C6	C19	O4
O2	OS	-0.34685	16	H23	HA	0.14334	52	C5	H1	C19	C20
C7	CA	0.10655	17	H24	H1	0.06698	53	O1	H11	C19	H2
C8	CA	0.03303	18	H25	H1	0.06698	54	C6	O2	O4	H26
C9	CA	-0.09343	19	H26	HO	0.42347	55	C6	H12	C20	H27
C10	CA	-0.10568	20	H27	H1	0.06407	56	C6	H13	C20	H28
C11	CT	0.04989	21	H28	H1	0.06407	57	O2	C7	C21	C22
C12	CT	-0.08559	22	H30	HA	0.14334	58	C7	C8	C21	H30
C13	CT	-0.08559	23	H31	HA	0.13931	59	C7	C24	C22	H31
C14	CA	-0.10170	24	H32	HA	0.13831	60	C8	C9	C23	C24
C15	CA	-0.09146	25	CL2	Cl	-0.05300	61	C8	Cl1	C23	H32
C16	CA	-0.17795	26	N1	NT	-0.92124	62	C9	C10	C24	Cl2
C17	CA	0.12483	27	H29	H	0.45770	63	C9	H15	C20	N1
O3	OS	-0.34486	28	S	SO	1.47504	64	C10	C11	N1	S
C18	CT	0.08274	29	O6	O	-0.65356	65	C10	C23	N1	H29
C19	CT	0.12162	30	O5	O	-0.65356	66	C11	C12	S	C25
H2	H1	0.05694	31	C25	CT	-0.36377	67	C11	C13	S	O6
O4	OH	-0.60228	32	H34	H1	0.11510	68	C11	C14	S	O5
C20	CT	0.22976	33	H35	H1	0.11510	69	C12	H16	C25	H34
C21	CA	-0.17795	34	H36	H1	0.11510	70	C12	H17	C25	H35
C22	CA	-0.09146	35					C12	H18	C25	H36
C23	CA	-0.09343	36					C13	H19	C	O

Table S7: Dihedral force field parameters of the modified cysteine residue CYS:EPI-7170 (CYE7). CYE7 dihedral parameters are applied using the improper dihedral section of the GROMACS CYE7 amino acid template.

[impropers]						
-C	N	CA	C	90.0	1.67360	2
N	CA	C	+N	160.888	2.71960	1
N	CA	C	+N	90.0	-1.0669	1
N	CA	C	+N	90.0	0.3138	2
N	CA	C	+N	90.0	0.2385	3
N	CA	C	+N	90.0	0.1046	4
N	CA	C	+N	90.0	-0.0460	5
-C	CA	N	H			
CA	+N	C	O			
C7	C9	C8	Cl1	180.00	4.60240	2
C7	C23	C24	Cl2	180.00	4.60240	2
C8	C10	C9	H15	180.00	4.60240	2
C8	C24	C7	O2	180.00	4.60240	2
C9	C23	C10	C11	180.00	4.60240	2
C10	C24	C23	H32	180.00	4.60240	2
C14	C16	C15	H22	180.00	4.60240	2
C14	C21	C22	H31	180.00	4.60240	2
C15	C17	C16	H23	180.00	4.60240	2
C15	C22	C14	C11	180.00	4.60240	2
C16	C21	C17	O3	180.00	4.60240	2
C17	C22	C21	H30	180.00	4.60240	2

Table S8: Added force field bond length parameters for modified cysteine residues CYE2 and CYE7. These parameters were added to the existing bond length parameters in the a99SB-*disp* force field.

[bondtypes]					
i	j	func	b0	kb	
OS	CA	1	0.13696	315140.0	; Amber99Sb-disp
CT	HO	1	0.10969	276650.0	; Amber99Sb-disp
NT	SO	1	0.16720	265350.0	; Amber99Sb-disp
NT	H	1	0.10100	363170.0	; Amber99Sb-disp
SO	CT	1	0.18080	195390.0	; Amber99Sb-disp
SO	O	1	0.14530	429030.0	; Amber99Sb-disp
CT	NT	1	0.14710	307110.0	; Amber99Sb-disp

Table S9: Added force field bond angle parameters for modified cysteine residues CYE2 and CYE7. These parameters were added to the existing bond angle parameters in the a99SB-*disp* force field.

[angletypes]						
i	j	k	func	th0	cth	
CT	SH	CT	1	99.240	503.750	; Amber99Sb-disp
CT	OS	CA	1	117.960	523.000	; Amber99Sb-disp
OS	CA	CA	1	119.200	582.410	; Amber99Sb-disp
CA	CT	CA	1	112.240	532.200	; Amber99Sb-disp
CA	CA	OS	1	119.200	582.410	; Amber99Sb-disp
CA	OS	CT	1	117.960	523.000	; Amber99Sb-disp
CT	CT	HO	1	109.560	388.280	; Amber99Sb-disp
OH	CT	HO	1	110.260	425.930	; Amber99Sb-disp
HO	CT	H1	1	108.460	328.030	; Amber99Sb-disp
CA	CA	Cl	1	118.800	585.760	; Amber99Sb-disp
CT	CT	NT	1	111.200	669.440	; Amber99Sb-disp
CT	NT	SO	1	116.550	525.510	; Amber99Sb-disp
CT	NT	H	1	109.500	418.400	; Amber99Sb-disp
NT	CT	H1	1	109.500	418.400	; Amber99Sb-disp
NT	SO	CT	1	101.970	537.230	; Amber99Sb-disp
NT	SO	O	1	107.430	595.800	; Amber99Sb-disp
SO	NT	H	1	109.600	374.890	; Amber99Sb-disp
SO	CT	H1	1	107.150	361.500	; Amber99Sb-disp
CT	SO	O	1	108.610	547.270	; Amber99Sb-disp
O	SO	O	1	120.050	615.880	; Amber99Sb-disp

Table S10: Added force field dihedral angle parameters for modified cysteine residues CYE2 and CYE7. These parameters were added to the existing dihedral angle parameters in the a99SB-*disp* force field.

[dihedraltypes]								
i	j	k	l	func	phase	kd	pn	
CT	OS	CT	CA	9	180.0	3.76560	0	; Amber99Sb-disp
CA	CA	OS	CT	9	180.0	3.76560	0	; Amber99Sb-disp
OS	CA	CA	Cl	9	180.0	15.16700	2	; Amber99Sb-disp
CA	CA	CA	Cl	9	180.0	15.16700	2	; Amber99Sb-disp
Cl	CA	CA	HA	9	180.0	15.16700	2	; Amber99Sb-disp
CT	CT	NT	SO	9	0.00	1.25520	3	; Amber99Sb-disp
CT	CT	NT	H	9	0.00	1.25520	3	; Amber99Sb-disp
CT	NT	SO	CT	9	0.00	13.10987	2	; Amber99Sb-disp
CT	NT	SO	O	9	0.00	13.10987	2	; Amber99Sb-disp
NT	SO	CT	H1	9	0.00	0.60436	3	; Amber99Sb-disp
SO	NT	CT	H1	9	0.00	1.25520	3	; Amber99Sb-disp
CT	SO	NT	H	9	0.00	13.10987	2	; Amber99Sb-disp
H1	CT	SO	O	9	0.00	0.60436	3	; Amber99Sb-disp
O	SO	NT	H	9	0.00	13.10987	2	; Amber99Sb-disp
CT	CT	CT	NT	9	0.00	0.65084	3	; Amber99Sb-disp
O	CT	CT	NT	9	0.00	0.65084	3	; Amber99Sb-disp
NT	CT	CT	H1	9	0.00	0.65084	3	; Amber99Sb-disp
H	NT	CT	H1	9	0.00	1.25520	3	; Amber99Sb-disp
